# Operando Laboratory‐Based Multi‐Edge X‐Ray Absorption Near‐Edge Spectroscopy of Solid Catalysts

**DOI:** 10.1002/anie.202209334

**Published:** 2022-10-26

**Authors:** Nina S. Genz, Antti‐Jussi Kallio, Ramon Oord, Frank Krumeich, Anuj Pokle, Øystein Prytz, Unni Olsbye, Florian Meirer, Simo Huotari, Bert M. Weckhuysen

**Affiliations:** ^1^ Inorganic Chemistry and Catalysis group Department of Chemistry Utrecht University Universiteitsweg 99 3584 CG Utrecht The Netherlands; ^2^ Department of Physics University of Helsinki P. O. Box 64 00014 Helsinki Finland; ^3^ Laboratory of Inorganic Chemistry Department of Chemistry ETH Zürich Vladimir-Prelog-Weg 1 8093 Zürich Switzerland; ^4^ Department of Physics Center for Materials Science and Nanotechnology University of Oslo P.O. Box 1048 0316 Oslo Norway; ^5^ Department of Chemistry University of Oslo P.O. Box 1033 0315 Oslo Norway

**Keywords:** Alloys, CO_2_ Hydrogenation, Heterogeneous Catalysis, Multielement Catalysts, X-Ray Absorption Spectroscopy

## Abstract

Laboratory‐based X‐ray absorption spectroscopy (XAS) and especially X‐ray absorption near‐edge structure (XANES) offers new opportunities in catalyst characterization and presents not only an alternative, but also a complementary approach to precious beamtime at synchrotron facilities. We successfully designed a laboratory‐based setup for performing operando, quasi‐simultaneous XANES analysis at multiple K‐edges, more specifically, operando XANES of mono‐, bi‐, and trimetallic CO_2_ hydrogenation catalysts containing Ni, Fe, and Cu. Detailed operando XANES studies of the multielement solid catalysts revealed metal‐dependent differences in the reducibility and re‐oxidation behavior and their influence on the catalytic performance in CO_2_ hydrogenation. The applicability of operando laboratory‐based XANES at multiple K‐edges paves the way for advanced multielement catalyst characterization complementing detailed studies at synchrotron facilities.

## Graphical abstract

Operando laboratory‐based multi‐edge X‐ray absorption near‐edge spectroscopy (XANES) offers possibilities for advanced catalyst characterization, as demonstrated for mono‐, bi‐, and trimetallic CO_2_ hydrogenation catalysts consisting of Ni, Cu, and Fe. Synergistic effects during catalyst reduction and their influence on catalytic performance were unraveled, guiding rational design of multielement solid catalysts for various applications.

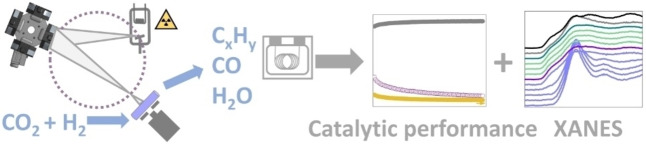

## Introduction

In catalysis research, the rational design of improved catalyst formulations is an ongoing challenge. Not the least because a rational catalyst design requires fundamental understanding of structure‐composition‐performance correlations of catalysts at work, which can only be deduced by applying appropriate characterization techniques in situ, under reaction conditions, or even operando, with simultaneous product analysis.[^1^, ^2^, ^3^, ^4^] This ambition also drives the continuous development of advanced X‐ray characterization techniques for catalysis research. Amongst those, laboratory‐based X‐ray absorption spectroscopy (XAS) and especially X‐ray absorption near‐edge structure (XANES) emerged as a highly promising approach for a fundamental characterization of functional materials. Although the achievable energy range limits the applicability of laboratory‐based XANES to certain chemical elements, there is great potential for, amongst others, its application to the study of transition metal‐based catalysts.[^5^, ^6^, ^7^]

XAS experiments are usually performed at synchrotron facilities enabling high resolution in both time and space.[^8^, ^9^, ^10^] However, beamtime at synchrotron facilities is precious and its availability is often limited, making the application for beamtimes very competitive. Synchrotron‐based XAS experiments are also limited in duration, hence, long‐term studies of several days or even weeks are difficult if not simply impossible and an appropriate selection of the catalysts has to be made beforehand. Therefore, only the most promising catalysts are chosen to be investigated in depth, excluding broad catalyst screening which is important for drawing more profound conclusions. Additionally, the need to transport air‐sensitive, toxic, or radioactive samples to the synchrotron facility can severely limit flexibility in studying such functional materials.[^7^, ^11^, ^12^, ^13^, ^14^, ^15^] These drawbacks can be overcome by performing XAS experiments in the laboratory, if the lower signal‐to‐noise (S/N) ratio, energy resolution, and longer measurement times of a laboratory‐based setup compared to a synchrotron facility are acceptable for the planned experiment(s). Under these restrictions, however, laboratory‐based XAS offers new opportunities in advanced catalyst characterization and presents an alternative as well as a complement to precious beamtime at synchrotron facilities.

Recent developments of laboratory‐based XAS and XES have been reviewed by Zimmermann et al.,^[14]^ and, more recently, by Malzer et al.,^[5]^ revisiting a century of laboratory‐based XAS with an outlook suggesting laboratory‐based XAS could develop into routine analysis. Indeed, so far several successful ex situ laboratory‐based XAS studies have been reported,[^12^, ^15^, ^16^, ^17^, ^18^, ^19^, ^20^, ^21^, ^22^, ^23^, ^24^, ^25^, ^26^] however, only a few in situ (under reaction conditions) experiments were published to date[^11^, ^27^, ^28^] and, to the best of our knowledge, operando (under real operating conditions with on‐line product analysis) laboratory‐based XAS in catalysis research has not yet been achieved. Given their complexity and importance in catalysis research, such operando spectroscopy experiments are, however, an essential step towards establishing XANES analysis as a (more) routine tool in the advanced catalyst characterization toolbox.

Operando laboratory‐based XAS in catalysis research can be applied in a multitude of interesting applications and one of those constitutes CO_2_ valorization. In view of continuously rising atmospheric CO_2_ concentration and increasing awareness of its associated consequences, CO_2_ valorization into chemicals and fuels became a highly relevant topic. Considering the ever‐growing global population associated with a further increasing energy demand but finite fossil feedstocks, using the greenhouse gas CO_2_ as reactant for producing value‐added products, e.g., methane, higher hydrocarbons as well as alcohols, such as methanol and ethanol, constitutes a promising starting point for reducing atmospheric CO_2_ concentrations.[^29^, ^30^, ^31^, ^32^] In this work, the thermo‐catalytic hydrogenation of CO_2_ over non‐noble, abundant, and relatively inexpensive metals, such as Ni, Fe, and Cu, has been studied. Ideally, CO_2_ can be captured from various sources, e.g., petrochemical and metallurgical industries or transportation, while the required H_2_ should be produced in a green, i.e., sustainable, way. To this end, renewable energy, such as wind or solar, can be used to produce green H_2_ via water electrolysis. Subsequently, thermo‐catalytic CO_2_ hydrogenation can be performed, whereby both the composition and structure of the catalysts as well as the reaction conditions determine the overall reaction pathway to varying sustainable chemicals and fuels. Therefore, one key to CO_2_ valorization is the design of efficient and selective solid catalyst materials.[^33^, ^34^]

In the last years, considerable progress has been reported for the thermo‐catalytic CO_2_ hydrogenation towards methane. In this context, Ni‐based catalysts are commonly desired due to their high activity and selectivity.[^35^, ^36^, ^37^, ^38^] However, for going one step further towards value‐added products besides methane, designing an optimal catalyst is still a challenging endeavor. For Ni‐based catalysts, a general approach to redirect the selectivity in CO_2_ hydrogenation towards C_2+_ products, is to suppress the strong hydrogenation ability and introduce active sites for both partial reduction of CO_2_ to CO and C−C coupling. One possibility to achieve this is to combine Ni with another metal that constitutes such active sites. Synergistically combining two metals represents an important strategy to tune the selectivity and activity of a catalytic reaction, by taking advantage of the selectivity of one metal and the activity of the second metal in a combined fashion.[^35^, ^39^, ^40^] Accordingly, bimetallic Ni−Fe or Ni−Cu catalysts seem to be promising candidates for tuning the selectivity in the CO_2_ hydrogenation towards value‐added products besides methane.

For bimetallic catalysts, improved performance is frequently ascribed to metal alloy formation, although it is not yet well‐understood how the two metals interact and boost the catalytic performance. Additionally, the optimal metal ratio, alloy phase, and required oxidation states are still under debate and different studies demonstrate the crucial role of those parameters for tuning the selectivity of a certain catalytic reaction.[^35^, ^39^, ^40^, ^41^, ^42^, ^43^, ^44^, ^45^] Moreover, this concept of a synergistic combination of several metals for a targeted tuning of the selectivity, can also be expanded to a trimetallic system, such as Ni−Cu−Fe, for the thermo‐catalytic CO_2_ hydrogenation. With increasing complexity of the catalyst systems, the appropriate characterization techniques become even more important as it is indispensable to gain insight into the role of all metals of importance for deducing reliable structure‐composition‐performance correlations. For this purpose, laboratory‐based XAS constitutes a powerful technique, especially when enabling operando studies at all absorption edges of the multielement catalyst materials.

Here, we report the first operando laboratory‐based XANES experiments at multiple K‐edges, more specifically XANES analysis at mono‐, bi‐, and trimetallic CO_2_ hydrogenation catalysts. The solid catalysts studied were Ni−Cu−Fe trimetallic, Ni−Fe and Ni−Cu bimetallic, as well as the corresponding monometallic counterparts, all supported on a SiO_2_ support. The detailed operando XANES studies provide new fundamental insights into synergistic effects during catalyst reduction and their influence on catalytic performance in CO_2_ hydrogenation. Hereby, we were able to unravel metal‐dependent differences in the reducibility and re‐oxidation behavior of the multi‐metal solid catalysts. It is important to note that a discussion of possible metal alloy formation in the multi‐metal catalysts is omitted in this study as this would require additional techniques, such as extended X‐ray absorption fine structure (EXAFS).

## Results and Discussion

### Design principles of the operando lab‐based setup

**Figure 1:**
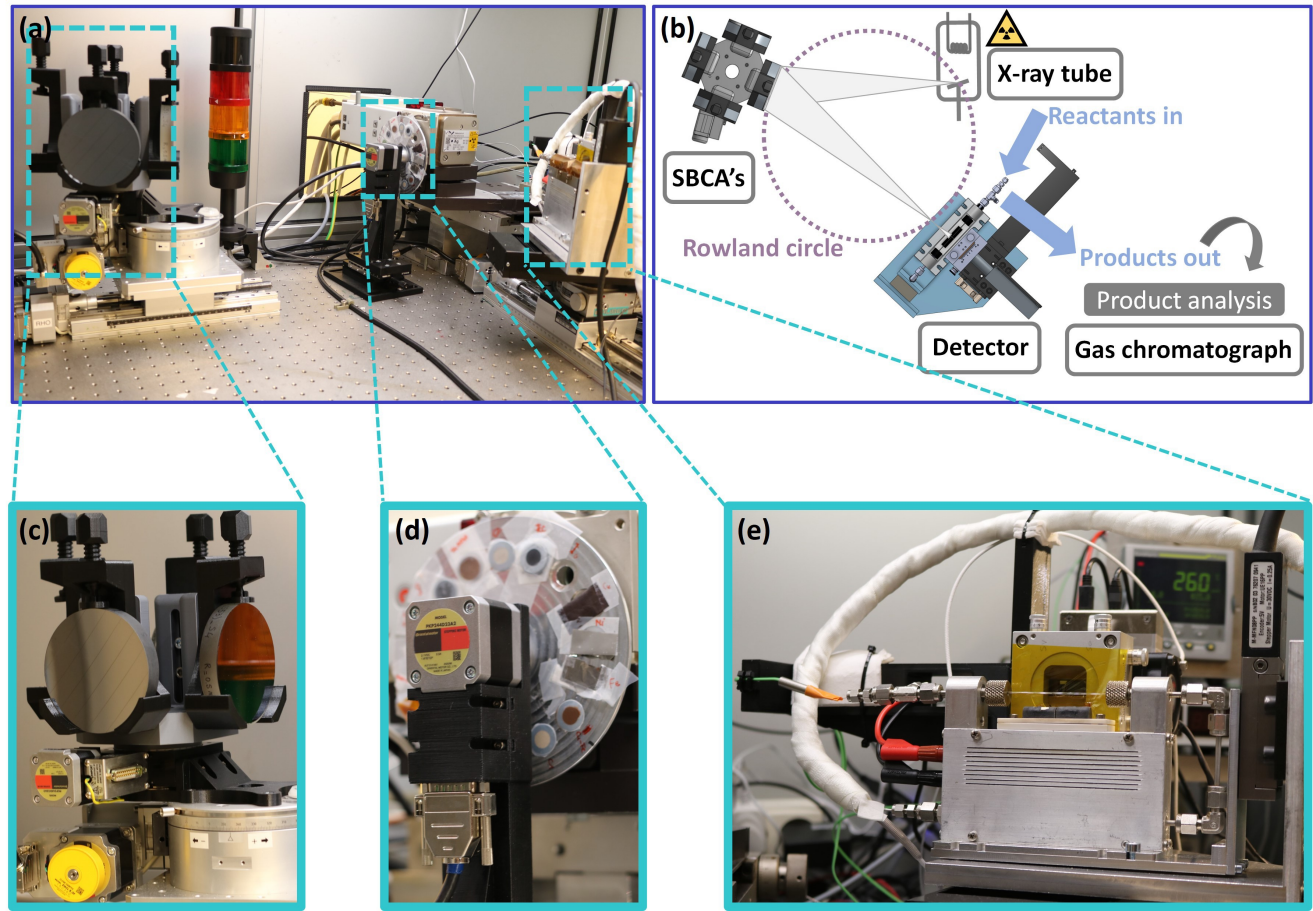
Photograph (a) and scheme (b) of the main components of the new operando laboratory‐based XANES setup, including the self‐designed motorized crystal exchanger (c), the ex situ sample wheel located in front of the X‐ray source (d), and the self‐designed motorized sample cell stage (e) for mounting the capillary cell in front of the slit upstream from the detector.

The main principle of the laboratory‐based XANES setup used is based on a Johann geometry, where the X‐ray source, the spherically bent crystal analyzers (SBCA's), and the detector are located on the Rowland circle with a radius of 0.5 m.^[6]^ With this laboratory‐based XANES setup in situ studies of Co‐based Fischer–Tropsch synthesis catalysts, including long‐term experiments up to 200 h, had already been performed successfully by our research groups.[^11^, ^27^] Based on these previous studies, we have now upgraded the former in situ setup towards a real operando laboratory‐based XANES setup, which now enables to measure quasi‐simultaneously (i.e., directly after each other under the same reaction conditions) at multiple K‐edges, including on‐line product analysis. This analytical upgrade comprises two main components, a self‐designed motorized crystal exchanger and a self‐designed motorized sample cell stage, whereon both the glass‐capillary‐based cell and the detector are located (Figure 1). Moreover, we now utilize the astigmatic imaging error of the SBCA's for generating a horizontal line‐focus of the X‐ray beam for optimizing the number of photons on the sample inside the capillary (see Supporting Information for more details, Figure S2). Briefly speaking, when using the Rowland circle geometry in combination with a point source at Bragg angles which deviate from 90°, spherically bent crystals generate two separated line images being perpendicular to each other.^[46]^ In order to utilize the horizontal rather than the vertical line‐focus, we therefore position the sample and the detector slightly outside the Rowland circle. The crystal exchanger, similar to that from Seidler et al.,^[15]^ offers four slots for mounting different SBCA's, which in turn allows for multiple K‐edge measurements within one in situ or operando experiment. The change from one K‐edge to the other, including all motor movements and change of the X‐ray tube settings takes less than 1 min and is highly reproducible (see Supporting Information for more details). All parameters for the different absorption edges are determined once before the operando experiment and subsequently set automatically at each edge change during the experiment. When employing various SBCA's, the precise alignment of the detector towards each of those crystals is indispensable. Therefore, the detector is located on the motorized sample cell stage, enabling both the movement along the Rowland circle during the energy scans and the alignment towards the selected crystal after each crystal exchange. The capillary cell is mounted in front of the slit, upstream from the detector, on this motorized sample cell stage. Hereby, measurements in transmission mode are realized. The vertical beam size is determined by the X‐ray source and constantly 0.8 mm, while the horizontal beam size depends on the Bragg angle and increases with decreasing Bragg angle. Here, the horizontal beam size ranges from 7.6 mm (for the largest Bragg angle of 78.7°) to 23 mm (for the lowest Bragg angle of 70.1°). All catalyst beds are 20 mm×0.96 mm. By using a slit with a constant opening of 10 mm between capillary cell and detector, possible leakage effects and different path lengths through the sample can be excluded. The catalyst powder is placed inside the glass capillary. During operando experiments at elevated temperature, the glass capillary tends to bend. In our setup capillary bending and correlated issues, e.g., moving of the capillary out of the line‐focus of the X‐ray beam, can easily be addressed by re‐adjusting the height of the capillary cell by moving the motorized sample cell stage. A further benefit of this motorized sample cell stage is the possibility to quickly change between ex situ and in situ/operando setups, something that is important e.g., for regular measurements of references and incident‐beam intensity. After the in situ/operando measurements, the capillary cell can be moved out of the X‐ray beam and the ex situ sample wheel, which is positioned in front of the X‐ray source, can be rotated towards a position of either reference pellets or metal foils. This enables ex situ reference measurements directly after finishing the in situ/operando measurements. Finally, for realizing operando experiments, an on‐line gas chromatograph is attached to the outlet of the capillary cell for simultaneous product analysis. Possible sources of systematic errors in measurements of the XANES are considered in the Supporting Information. Most importantly, the mechanical precision and negligible heat load results in high precision in the energy calibration and beam stability, even better than what is typically encountered at a synchrotron facility.

### Operando X‐ray absorption spectroscopy of solid catalysts at multiple K‐edges

Each operando XANES experiment starts with an in situ pre‐reduction of the catalysts. Subsequently, the gas atmosphere is switched towards CO_2_ hydrogenation conditions for investigating changes during catalytic reaction. During these experiments, XANES analysis is performed at the relevant K‐edges of the corresponding solid catalysts. Each scan at the Fe and Cu K‐edge is conducted with a step size of 0.5 eV and a radiation time of 5 s per step in the energy range of 7.085–7.175 keV and 8.94–9.03 keV, respectively. A scan at the Ni K‐edge is conducted between 8.315 and 8.405 keV with a step size of 0.5 eV and a radiation time of 10 s per step. Hence, the time to collect one XANES is 15 min for the Fe and Cu K‐edge and 29.3 min for the Ni K‐edge. For all experiments, the measurement at multiple absorption edges is performed in the order from Ni to the other element(s) and further to Ni, e.g., for the trimetallic catalyst in the order Ni−Cu−Fe−Ni at each temperature step. Moreover, XANES are collected isothermally, i.e., only after reaching the next constant temperature, to allow a comparison of all absorption edges without a temperature drift during the acquisition. The holding time at each temperature step is adjusted depending on the number of elements of the catalyst. The longest holding time of 236 min per step is conducted for the trimetallic catalyst, while it is 194 min for all bimetallic catalysts and 148 min, 46 min, and 44 min for the monometallic Ni, Cu, Fe catalyst, respectively (Figure S3). To increase the S/N ratio by factor of 2, the number of XANES, and hence, the holding times at 520 °C during reduction and 400 °C during CO_2_ hydrogenation, being considered as most important reaction steps, are increased by factor of 4. For all catalysts under study, the total metal loading and the metal ratio were kept constant at about 6 wt % and 1, respectively, and the average metal nanoparticle size as determined by transmission electron microscopy studies is in the range 0.7–2.5 nm, except for Cu/SiO_2_ comprising a few agglomerates (see Supporting Information for more details). In this work, we have performed for the first time operando laboratory‐based XANES of multi‐metal catalyst materials quasi‐simultaneously at all, up to three, K‐edges. In what follows, we will first discuss the operando XANES of the multi‐metal catalyst materials, and make comparison with the monometallic counterparts. Second, we focus on the correlation between XANES and catalytic performance in CO_2_ hydrogenation.

### Operando XANES of multi‐metal catalyst materials

**Figure 2:**
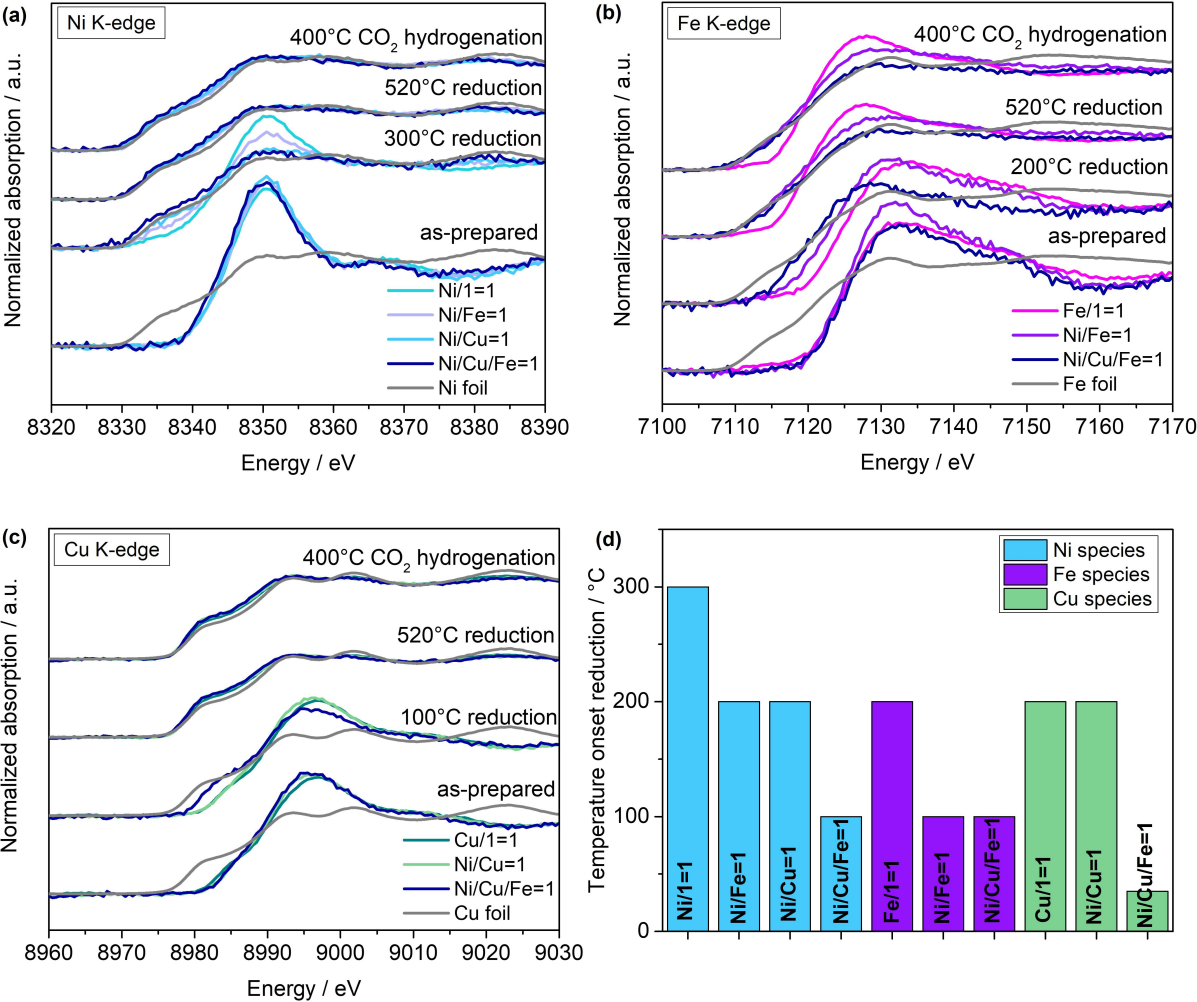
Comparison of selected operando XANES data recorded during reduction (H_2_ : He=1 : 1, 1 bar) and catalytic CO_2_ hydrogenation (H_2_ : CO_2_ : He=4 : 1 : 5) for trimetallic Ni−Cu−Fe/SiO_2_ (Ni/Cu/Fe=1), bimetallic Ni−Fe/SiO_2_ (Ni/Fe=1) and Ni−Cu/SiO_2_ (Ni/Cu=1), and corresponding monometallic catalysts (Ni/1=1, Fe/1=1, Cu/1=1) at the Ni (a), Fe (b), and Cu (c) K‐edge. Significant differences in the reducibility of the multi‐metallic compared to corresponding monometallic catalysts are highlighted by comparing the temperatures for the reduction onset (d) and the XANES at 300, 200, 100 °C, respectively, during reduction (a–c).

It is evident from the XANES in Figures 2, S19 at the Ni and Fe K‐edge that the formation of the bimetallic Ni−Fe/SiO_2_ catalyst induces a change in the reducibility of both the Ni and the Fe metal nanoparticles compared to those present in the corresponding monometallic counterparts. The reducibility of the Ni species is facilitated, i.e., possible at lower temperature, compared to the monometallic Ni/SiO_2_ catalyst (Figures 2a, d, S19). The Ni species in the Ni−Fe/SiO_2_ catalyst stay in the initial state until 200 °C and get reduced between 200 and 300 °C, while the reduction onset is at 300 °C in the monometallic counterpart. Interestingly, for the Fe species, the formation of a bimetallic Ni−Fe/SiO_2_ catalyst also facilitates the reducibility, but additionally hinders the re‐oxidation of the Fe species when changing the gas atmosphere from reducing (H_2_/He) towards CO_2_ hydrogenation (H_2_/CO_2_/He) conditions that is seen for the monometallic catalyst (Figures 2b, d, S19). For the Fe species, the reduction already starts from 100 °C and the species are continuously reduced until reaching 400 °C, whereas in the monometallic Fe/SiO_2_ the reduction starts only above 200 °C. Moreover, the reduction of the Fe species in Ni−Fe/SiO_2_ proceeds more progressively and towards a more reduced state compared to the corresponding Fe/SiO_2_ catalyst (Figure 2b, S19). These distinct changes in the reducibility and the re‐oxidation behavior indicate a synergistic effect between Ni and Fe for the bimetallic Ni−Fe/SiO_2_ catalyst. An improved reducibility of supported Ni‐based bimetallic catalysts has previously been reported for Ni_3_Fe/Al_2_O_3_ (17 wt %),^[42]^ NiMn(0.25)/silica‐modified γ‐Al_2_O_3_ (17 wt %),^[35]^ Ni_3_Fe_3_/CeO_2_ (2.9 wt %) and Ni_3_Fe_3_/CeO_2_ (2 wt %)^[40]^ catalyst systems. However, the reason for such an improved reducibility is not yet consistently elucidated. While for the CeO_2_‐supported bimetallic catalysts, the facilitated hydrogen spillover by metallic Ni is ascribed to the improved reducibility of the Fe species, for the Ni−Mn system, the presence of a Ni−Mn‐oxide phase is claimed to decrease the Ni‐support interactions, yielding an improved reducibility. However, the observed progressive reduction of the Fe species starting earlier than the reduction of the Ni species excludes an improved reducibility due to the facilitated hydrogen spillover of metallic Ni as it was postulated for low‐loaded CeO_2_‐supported Ni−Fe catalysts.^[40]^ Moreover, once reduced, both the Ni and Fe species stay in this reduced state also during the complete subsequent CO_2_ hydrogenation (Figures 2a, b, S18)

When combining Ni and Cu instead of Ni and Fe, we observe a similar change in the reducibility of the Ni species. Compared to the monometallic Ni/SiO_2_, the XANES at the Ni K‐edge for the bimetallic Ni−Cu/SiO_2_ catalyst indicate a distinctly facilitated reducibility (Figures 2a, d, S20). Surprisingly, for the Cu species, no distinct influence of the formation of a bimetallic Ni−Cu system can be revealed (Figures 2c, d, S20). The reduction of the Ni and Cu species in the bimetallic Ni−Cu/SiO_2_ catalyst starts at the same temperature, namely ∼200 °C. These observations again indicate a synergistic effect between Ni and Cu.

By combining Ni with Fe and Cu in a trimetallic catalyst formulation, similar to the bimetallic systems, a synergistic effect between Ni, Fe, and Cu can be revealed. From Figures 2d and S21, it can be concluded that the reduction of the Ni and Fe species starts at the same temperature, from about 100 °C, while that of the Cu species starts even earlier, i.e., below 100 °C. Hence, the synergistic effects between Ni, Fe, and Cu, facilitate the reducibility of all three metals, that is, the reduction starts at lower temperatures compared to corresponding mono‐ and bimetallic catalysts.

### Correlation between XANES and catalytic performance in CO_2_ hydrogenation

**Figure 3:**
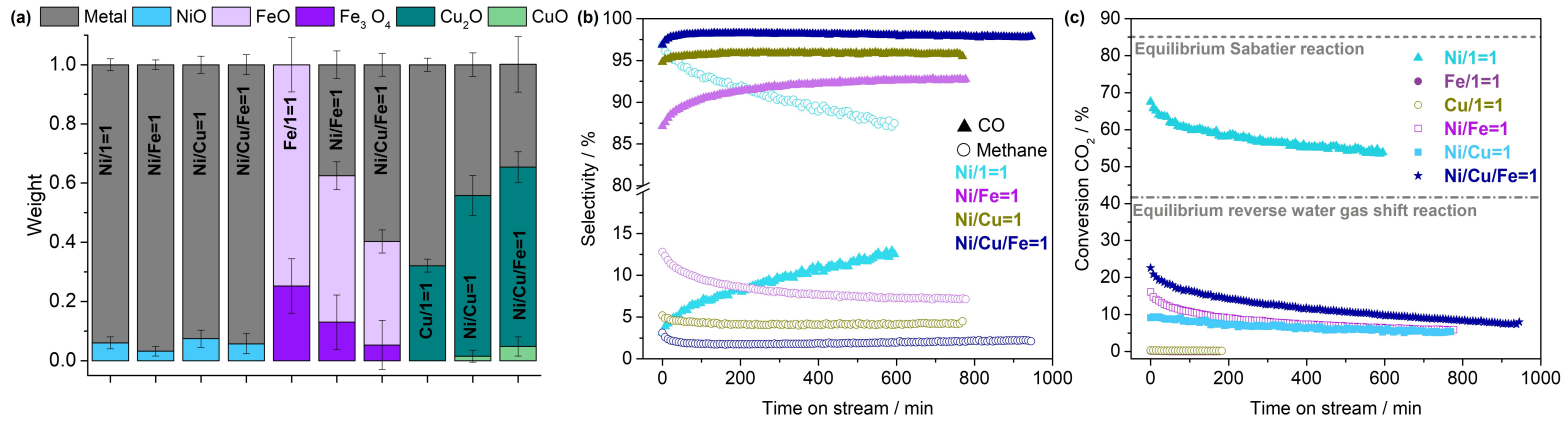
Sample composition based on LCF of the XANES at 400 °C during catalytic CO_2_ hydrogenation (H_2_ : CO_2_ : He=4 : 1 : 5, 1 bar, GHSV=4.1 10^4^ h^−1^) (a) together with selectivity towards the main reaction products (b, CO as filled symbols, methane as empty symbols) and CO_2_ conversion (c) for the trimetallic Ni−Cu−Fe/SiO_2_ (Ni/Cu/Fe=1), bimetallic Ni−Fe/SiO_2_ (Ni/Fe=1) and Ni−Cu/SiO_2_ (Ni/Cu=1), and corresponding monometallic catalysts (Ni/1=1, Fe/1=1, Cu/1=1) as function of time on stream at 400 °C. R‐factors from LCF are the following from left to right: 0.0036, 0.0023, 0.0074, 0.0113, 0.0629, 0.0066, 0.0049, 0.0008, 0.0017, 0.0040.

By combining Ni with Fe and/or Cu, we aim at tuning the selectivity in the catalytic CO_2_ hydrogenation compared to the monometallic Ni/SiO_2_ catalyst. Hence, it is important to correlate the revealed synergistic effects during reduction in the multi‐metal catalysts directly with the simultaneously conducted product analysis. To this end, the product selectivity as function of time on stream at 400 °C during catalytic CO_2_ hydrogenation and the results from linear combination fitting (LCF) of the corresponding XANES are depicted in Figure 3. Importantly, prior to LCF, significant changes of the XANES with time on stream at 400 °C during CO_2_ hydrogenation were excluded (Figure S23). It is evident that the major phase of the Ni species is metallic Ni (∼93–97 %) for the monometallic, both bimetallic, and the trimetallic systems. However, the product selectivity changes drastically, indicating a distinct influence of the synergistic effects in the multi‐metal catalyst materials on their catalytic performance. While the monometallic Ni/SiO_2_ catalyst shows a high selectivity towards methane (∼96.9–87.5 %) and a continuous deactivation, both bimetallic catalysts tune the selectivity towards CO and additionally diminish the deactivation. The selectivity towards CO is superior for the Ni−Cu/SiO_2_ (∼94.8–95.5 %) compared to corresponding Ni−Fe/SiO_2_ catalyst (∼87.1–92.8 %). The synergistic effect between Ni and Fe induces a significant contribution of Fe^0^ species (≈38 %), while the major phase can still be assigned to Fe^II^ species (∼50 %) and a minor contribution of Fe^II,III^ species (∼13 %). Considering the marginal activity of the monometallic Fe/SiO_2_ catalyst, being composed of Fe^II^ and Fe^II,III^ species whereby the Fe^II^ species contribute with ≈75 %, we can conclude that most likely Fe^0^ species are essential for the catalytic performance in CO_2_ hydrogenation. Contrary to the Ni−Fe/SiO_2_, for the Ni−Cu/SiO_2_ catalyst, the synergistic effect between Ni and Cu decreases the contribution of Cu^0^ species (from ∼68 to 44 %), while increasing that of Cu^I^ species (from ∼32 to 54 %) compared to the corresponding monometallic Cu/SiO_2_ catalyst. As the monometallic Cu/SiO_2_ catalyst shows only marginal CO_2_ conversion, these results indicate Cu^I^ rather than Cu^0^ species being essential for the catalytic performance in CO_2_ hydrogenation. This conclusion is in accordance with a previous study on Ni−Cu/Al_2_O_3_ catalysts where a decrease in CO_2_ methanation activity was correlated with the increased surface enrichment of Cu metal, being claimed as inactive for this reaction.^[39]^

Comparing the selectivity towards the main reaction products for the trimetallic Ni−Cu−Fe/SiO_2_ with both bimetallic systems and the monometallic Ni/SiO_2_ reveals a further shift of the product selectivity towards CO (96.9–97.9 %). Moreover, similar to the bimetallic systems, the deactivation with time on stream is minor compared to the Ni/SiO_2_ catalyst (Figure 3b, c). At 400 °C during catalytic CO_2_ hydrogenation, LCF reveals a contribution of the metallic species for Ni, Fe, and Cu (Figure 3a). Interestingly, the contribution of the metallic phase decreases from Ni to Fe, and further to Cu (94 %, 60 %, 35 %). A significant contribution of Fe^II^ (∼35 %) and Cu^I^ (∼61 %) species can additionally be assigned. While the trimetallic formation does not affect the Ni species, i.e., metallic Ni is still the major phase, the contribution of Fe^0^ species and that of Cu^I^ species significantly increased. Our hypothesis that Fe^0^ and Cu^I^ species are essential for the catalytic performance in catalytic CO_2_ hydrogenation is in accordance with the increased contribution of Cu^I^ (∼61 %) and Fe^0^ (∼60 %) species and the increased CO selectivity of the trimetallic catalyst.

Moreover, catalytic CO_2_ hydrogenation over Ni−Fe/SiO_2_ yields small amounts of C_2+_ products while over the trimetallic Ni−Cu−Fe/SiO_2_ small amounts of ethylene are produced. This observation can be explained by the C−C coupling ability of Fe. Compared to the bimetallic Ni−Fe/SiO_2_ catalyst, the average yield of ethylene during catalytic CO_2_ hydrogenation at 400 °C over the trimetallic Ni−Cu−Fe/SiO_2_ catalyst decreased from 0.85 to 0.27 10^−5^ mol g^−1^ s^−1^, which can be correlated with the decreased ratio of Fe^II^/Fe^0^ species (Figure S29).

Overall, the formation of bi‐ and trimetallic systems induces a distinct change in product selectivity, while CO_2_ conversion is decreased (Figure 3b, c). This influence can directly be correlated with the synergistic effects between Ni and Fe and/or Cu. Based on this synergistic combination, the idea of multi‐metal systems is to benefit from the high activity of Ni and the selectivity of Fe and/or Cu in a combined fashion. Hence, using operando laboratory‐based XANES studies, we can transition from a “trial and error” towards an “educated guess” approach for the design of new or improved solid catalysts.

The present study demonstrates, that only if we can conduct such type of experiments, i.e., operando XANES at multiple K‐edges, we can deduce a fundamental understanding of multielement solid catalysts, being indispensable for a rational design of improved catalysts. Even though the presented study could have been done also at a synchrotron facility, the possibility to perform such wide set of operando XANES experiments in a laboratory offers new opportunities for detailed catalyst characterization. For multielement solid catalysts, a multitude of different parameters needs to be optimized, which in turn costs precious time at a synchrotron facility. In this context, laboratory‐based XAS is especially powerful as it holds the promise of being used routinely ‐ it can therefore become an analytical method for not only regularly checking design and success of the synthesis method to prepare the multielement catalysts, but also to validate catalyst performance via operando spectroscopy experiments.

## Conclusion

We have successfully developed an operando laboratory‐based XANES setup, which allows to measure quasi‐simultaneously solid catalysts at multiple K‐edges. The experimental setup has been used to investigate the catalytic hydrogenation of CO_2_ over mono‐, bi‐, and trimetallic solid catalysts, consisting of Ni, Fe, Cu, Ni−Fe, Ni−Cu, and Ni−Cu−Fe metal nanoparticles supported on SiO_2_. This method allowed to unravel metal‐dependent differences in the reducibility and re‐oxidation behavior of these multielement solid catalysts, being induced by the synergistic effects between the different metals. The formation of a bimetallic catalyst increases the reducibility of the Ni and the Fe species, while that of the Cu species remains unaffected. However, for the trimetallic Ni−Cu−Fe/SiO_2_ catalyst, the reducibility of all species is facilitated. Moreover, the synergistic effects between Ni and Fe and/or Cu unambiguously tune the selectivity in CO_2_ hydrogenation from methane towards CO, which is an important intermediate for the formation of C_2+_ products via e.g., Fischer–Tropsch synthesis. Accordingly, we can conclude that the addition of so‐called green metals that are non‐toxic, abundant, and relatively inexpensive, which holds for Fe and Cu, to Ni‐based catalysts constitutes a promising approach towards designing solid catalysts for CO_2_ valorization. Further improvement in terms of catalyst activity is the next research task and we expect laboratory‐based XANES to play a crucial role in such studies, where a large parameter space of catalyst composition and structures needs to be explored. As the presented setup is designed for experiments in transmission mode, the concentration of the absorbing element likewise the absorbance of the matrix constitutes crucial parameters for the applicability. In case of highly‐diluted samples such as metal‐exchanged zeolites, metal‐loaded MOF catalysts, or nm‐scale thin films, XAS can be conducted in fluorescence mode.^[47]^ It is in principle possible to perform EXAFS measurements, but they generally require an order of magnitude longer measurement times. Hence, in order to balance between a reasonable measurement time and gained information yield, it could be a future approach to perform EXAFS only at certain steps during the operando experiment. Moreover, the applicability of operando XANES is not limited to thermo‐catalysis and can likewise be applied to e.g., electrocatalysis by properly designed reaction cells.

## Experimental Section

Experimental procedures and supplementary data, e.g., characterization data of the catalysts, are described in the Supporting Information.

## Conflict of interest

The authors declare no conflict of interest.

1

## Supporting information

As a service to our authors and readers, this journal provides supporting information supplied by the authors. Such materials are peer reviewed and may be re‐organized for online delivery, but are not copy‐edited or typeset. Technical support issues arising from supporting information (other than missing files) should be addressed to the authors.

Supporting Information

## Data Availability

The data that support the findings of this study are available from the corresponding author upon reasonable request.
