# Supplementary material for: Operando Laboratory‐Based Multi‐Edge X‐Ray Absorption Near‐Edge Spectroscopy of Solid Catalysts
Source: Angew Chem Int Ed Engl. 2022 Oct 26;61(48):e202209334. doi: 10.1002/anie.202209334 (PMC9828672; doi:10.1002/anie.202209334)
Supplement: Supplementary file 1 — Supporting Information [file ANIE-61-0-s001.pdf]

Angewandte Chemie
Eine Zeitschrift der Gesellschaft Deutscher Chemiker

Supporting Information

Operando Laboratory-Based Multi-Edge X-Ray Absorption Near-Edge Spectroscopy of Solid Catalysts

*N. S. Genz, A.-J. Kallio, R. Oord, F. Krumeich, A. Pokle, Ø. Prytz, U. Olsbye, F. Meirer, S. Huotari, B. M. Weckhuysen**

Table of Contents

Table of Contents ... 2
Experimental Procedures ... 2
Catalyst Synthesis ... 2
Catalyst Characterization ... 2
X-ray Diffraction ... 2
Transmission Electron Microscopy ... 3
Operando Laboratory-based X-ray Absorption Near-Edge Structure ... 3
Thermodynamic Calculations ... 5
Results and Discussion ... 6
Standard Catalyst Characterization ... 6
Catalyst Characterization by Operando Laboratory-based XANES ... 19
Possible Systematic Errors of Laboratory-based XAS ... 19
Estimation of the Energy Resolution at the Different Absorption Edges ... 19
Comparison of Metal Foils Measured in the Laboratory-based Setup and at a Synchrotron Facility ... 20
Operando XANES Results of the Monometallic Catalysts ... 21
Operando XANES Results of the Multi-Metal Catalyst Materials ... 22
Average Oxidation States of the Species of the Supported Catalyst Materials ... 24
Degree of Re-oxidation during CO_2 Hydrogenation Conditions ... 26
Linear Combination Fitting ... 27
Comparison between Reduction and CO_2 Hydrogenation ... 31
Additional Catalytic Performance Data ... 32
References ... 32
Author Contributions ... 33

Experimental Procedures

Catalyst Synthesis

All catalyst materials were prepared using the incipient wetness impregnation (IWI) technique. High-purity grade silica gel (Davisil Grade 643, pore size 150 Å, 200-425 mesh, Sigma Aldrich) was used as support material. The required amount of metal precursor ($Ni(NO_3)_2$ x 6 H_2O (Sigma Aldrich, 99.999%), $Fe(NO_3)_3$ x 9 H_2O (Sigma Aldrich, ≥ 99.999%), $Cu(NO_3)_2$ x 2.5 H_2O (Sigma Aldrich, ≥99.99%)) was dissolved in water, whereby the volume of water was adjusted to that of the pore volume of the silica support (V_{pore} = 1.15 $cm^3\,g^{-1}$). The IWI approach was conducted similar to that reported by Winter *et al.*[1] with some slight modifications. After adding the aqueous precursor solution dropwise to the silica support, the impregnated catalysts were dried at room temperature for at least 24 h and subsequently calcined at 450°C. The weight loading of all as-prepared catalyst materials was confirmed by inductively coupled plasma-optical emission spectroscopy (ICP-OES) measurements.

Catalyst Characterization

X-ray Diffraction

Ex situ X-ray diffraction (XRD) patterns of all as-prepared catalyst materials were recorded on a Bruker D2 PHASER instrument with Co K_α radiation (1.78897 Å) in the 2θ range of 10-85°, step size of 0.02° and a radiation time of 10 s per step.

Transmission Electron Microscopy

(Scanning) transmission electron microscopy ((S)TEM) measurements were performed in two different approaches. In order to gain first insights into particle sizes, TEM measurements of the as-prepared and spent catalyst materials were conducted on a JEOL JEM-2100F microscope with a Schottky field emission gun (FEG), which was operated at an acceleration potential of 200 kV. The catalyst powders were sonicated in an ethanol solution and drop-casted on a lacey carbon support film TEM Cu grid. TEM images were acquired using a Gatan Orius 200D CCD camera and data evaluation was performed with the Gatan Microscopy Suite (GMS) 3 software. Moreover, high-resolution (S)TEM measurements were performed to further unravel the particle sizes and particle size distributions of all supported catalyst materials. To this end, (S)TEM measurements combined with energy-dispersive X-ray (EDX) spectroscopy of the as-prepared and spent trimetallic Ni-Cu-Fe/SiO_2 catalyst were performed on a Talos F200X microscope (Thermo Fisher Scientific) with a high brightness field emission gun operated at an acceleration potential of 200 kV. The EDX system attached to this microscope consists of 4 silicon drift detectors (SDD). While (S)TEM investigations of the trimetallic catalyst were performed on a F200X microscope, those of the bi- and monometallic catalysts were performed on an aberration-corrected, dedicated STEM microscope, a HD-2700CS (Hitachi), operated at an acceleration potential of 200 kV (cold field emitter). A probe corrector (CEOS) that is incorporated in the microscope column between the condenser lens and the probe-forming objective lens provides a resolution below 0.1 nm.[2] Different detectors can be selected for imaging in bright field (BF) and dark field mode ((high-angle) annular dark field ((HA)ADF)). In HAADF-STEM, the image is generated with incoherently scattered electrons resulting in an intensity strongly increasing with the atomic number (Z-contrast). Furthermore, a secondary electron detector is installed inside the column of the HD-2700CS microscope allowing to study the sample morphology. Hence, secondary electron images were recorded not only for all mono- and bimetallic but also for the trimetallic catalyst on this microscope. The images (1024 × 1024 pixels) were recorded with frame times between 10 and 20 s. Analytical investigations done with an energy-dispersive X-ray spectrometer (EDXS, EDAX) attached to the microscope column confirmed the presence of the corresponding metals in the nanoparticles For all high-resolution (S)TEM measurements, the catalyst powders were dispersed in ethanol and a few drops of the suspension were deposited onto a perforated carbon foil supported on a Cu or Mo grid depending on the catalyst composition, i.e., catalysts containing Cu were deposited on a Mo grid. After evaporation of the ethanol, the grid was mounted on the single tilt holder of the respective microscope. Particle size distributions were determined with the software ImageJ.

Operando Laboratory-based X-ray Absorption Near-Edge Structure

In this work, an Ag-tube is used as polychromatic X-ray source (source size 0.4 x 0.8 mm^2), the detector is an Amptek silicon drift detector (SDD), and the employed spherically bent crystal analyzers (SBCA's) are Si(531), Si(551), Si(553) for the Fe, Ni, and Cu K edge (7112 eV, 8333 eV, 8979 eV), respectively. All crystals possess a surface diameter of 100 mm and a bending radius of 0.5 m. During the measurement, both the selected SBCA and the detector are moving in order to enable the energy scan across the specific K edge, while the X-ray source is kept fixed in position. Operando laboratory-based X-ray absorption near-edge structure (XANES) was performed at the Ni K edge with an accelerating potential of the Ag-tube of 10 kV and a current of 2 mA, while the tube settings were 20 kV and 40 mA for the spectra at the Fe and Cu K edge. To increase the S/N ratio, all depicted spectra are averaged spectra. For XANES at the Ni K edge, four single scans were averaged to the resulting spectra, while for the Fe and Cu-K edge, two scans were averaged to the resulting spectra. This procedure was conducted for all temperature steps except for 520°C during reduction and 400°C during CO_2 hydrogenation. Here, the holding time was four times longer, resulting in 16 and 8 scans for the Ni and Fe/Cu K edge which were used to average. XANES data analysis was conducted with MATLAB R2019b and Athena 09.26.[3] All depicted contour plots (Figures S18, S19 e, f, S20 e, f, S21 b, d, f) are constructed from the area-normalized XANES spectra and the scale bar refers to arbitrary units.

The catalyst powder was located inside the glass capillary (inner diameter 0.96 mm) and two quartz wool plugs were inserted to fix the catalyst in position. To control the actual reaction temperature, a K-type thermocouple was inserted into the capillary, into the catalyst bed. A total gas flow of 10 mL min^{-1} was applied during both reduction and CO_2 hydrogenation. All experiments were conducted at atmospheric pressure.

Analysis of the gaseous products during the operando XANES experiments was performed using a gas chromatograph (GC, CompactGC, Interscience). The hydrocarbon products were detected by the flame ionization detector (FID), while H_2, CO, CO_2, O_2, and N_2 were detected by the thermal conductivity detector (TCD). The GC was equipped with a Rt-QBond (10 m x 0.32 mm) for the FID channel and a ShinCarbon ST Packed Column (2 m x 0.16") with a Hayesep Q pre-column (0.5 mx 1/16") for the TCD channel.

Linear combination fitting (LCF) of the XANES at 400°C during CO_2 hydrogenation for the various K edges was performed using the software Athena 09.26.[3] To this end, several reference spectra were considered by using a combinatorial fit approach. Hereby, the weights were forced to range between 0 and 1, while the sum was forced to be 1. For the XANES at the Ni K edge, NiO and Ni foil were employed as reference spectra, while for the spectra at the Fe and Cu K edge, Fe_2O_3, Fe_3O_4, FeO, Fe foil and CuO, Cu_2O, Cu foil, were considered.
In order to determine the average oxidation state of the species of the supported catalysts, the position of the first maximum of the first derivative of the XANES was used. This approach was conducted with MATLAB R2019b. A separate error estimation was conducted for each temperature step for each catalyst. First, the maximum of the first derivative was chosen based on the comparison with the

metal foil. Especially for the spectra with lower S/N ratio, additional local extrema were visible. Accordingly, the next meaningful neighboring local extremum, either towards lower or higher energy, was considered for estimating the error based on the energy difference between these two local extrema for each spectrum. For all XANES, the error ranged between 0.5 and 1.5 eV depending on the S/N ratio of the corresponding temperature step and the number of averaged spectra.

Images of the X-ray beam with a position sensitive detector (Advacam Minipix, pixel size 55 µm x 55 µm) at all absorption edges are collected and depicted in Figure S1. These images demonstrate the increasing beam size in horizontal dimension with decreasing Bragg angle. In this work, a horizontal slit of 10 mm in width was used to limit the maximum horizontal beam size to 10 mm.

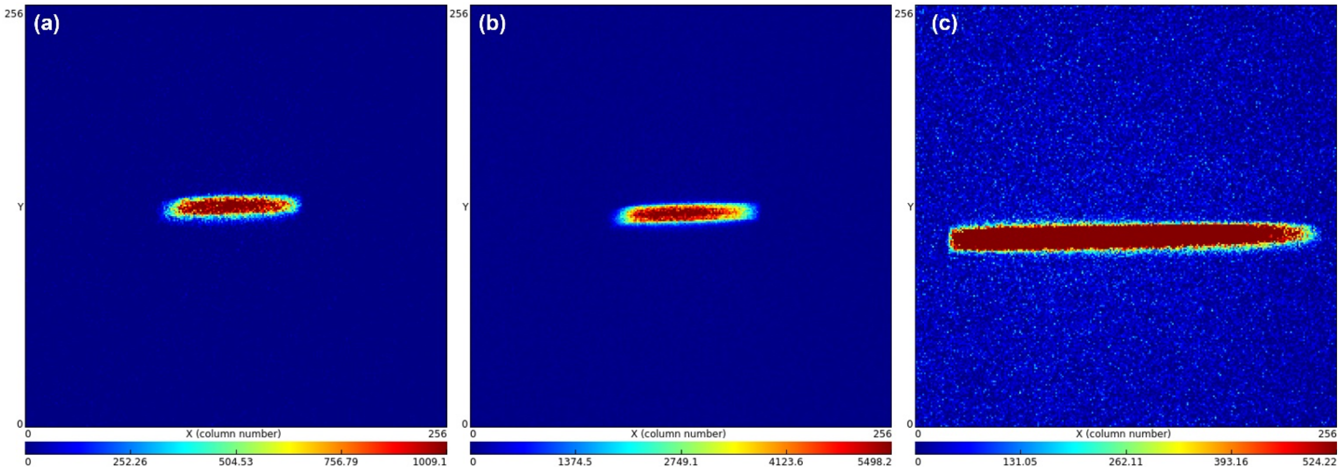

Figure S1. Images of the beam size at the Ni K edge at position 8315 eV (a), at the Cu K edge at position 8940 eV (b), and at the Fe K edge at position 7090 eV (c). A position sensitive detector was used to collect the beam size images. The image area in all three cases is 14 mm x 14 mm.

In this work, the horizontal line-focus of the X-ray beam was used, however, when using SBCA's and a Rowland circle geometry, it is possible to differentiate three cases: (a) a horizontal line-focus of the X-ray beam when positioning the sample and detector outside the Rowland circle at the position of the sharpest line-focus (as it is the case in the here presented study), (b) a circular beam focus if the sample and detector are positioned outside the Rowland circle but not at the position of the sharpest line-focus, and (c) a vertical line-focus of the X-ray beam when positioning the sample and detector on the Rowland circle. Considering the constant vertical beam size of 0.8 mm being determined by the X-ray source in the here presented setup and the horizontal beam size being dependent on the Bragg angle and ranging between 7.6 and 23 mm, it is important to note that the vertical beam spacing cannot be tuned. Comparing the overlapping area (yellow) between X-ray beam (green) and sample inside the glass capillary (blue) of all three cases reveals that case (a) results in the largest overlapping area. Considering the vertical beam size of 0.8 mm and a horizontal beam size of 23 mm in (a) results in a maximal overlapping area between X-ray beam and sample, and hence, an increased intensity compared to (c). Accordingly, the horizontal line-focus used in the here presented experiments enables that all photons can in principle interact with the sample. Of course, the actual amount of photons interacting with the sample depends on the cross section, but this holds for all three cases. Based on Figure S2, it becomes evident that the position of the sample and detector outside the Rowland circle with the sharpest horizontal line-focus of the X-ray beam is the most beneficial geometry when using SBCA's and a capillary reaction cell.

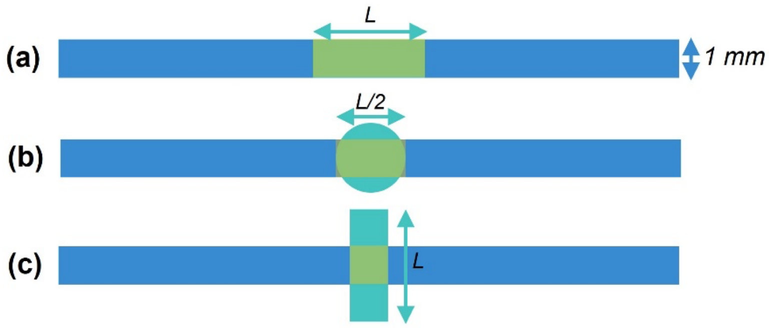

Figure S2. Scheme of three possible beam geometries (green) on the glass capillary (blue) and corresponding overlapping area (yellow) between X-ray beam and catalyst bed inside the glass capillary. The outer thickness of the capillary is 1 mm, and L is the size of the line-focus (e.g., 7.6 – 23 mm in our case without slitting).

Essential for the operando XANES experiments is the determination of the sharpest line-focus for all absorption edges. This procedure is performed prior to the operando experiments by locating a metal wire into the capillary cell in front of a position sensitive detector. While moving the sample stage from the position at the Rowland circle towards positions further outside, the signal is compared until the sharpest horizontal line-focus is found. It is ensured that the line-focus is optimized for all absorption edges and saved in the measurement macro, allowing for an automated realizing of the sharpest horizontal line-focus after each edge change.

Moreover, before starting the operando experiments, an energy calibration for all absorption edges is performed using standard metal foils. Additionally, all parameters for the different absorption edges, e.g., ROI, crystal exchanger motor positions, crystal spacings, optimal position for the sharpest line-focus, and X-ray tube settings, are determined and set into the measurement macro. Hence, whenever the absorption edge is changed during the experiment, the determined edge-specific parameters are automatically applied. The only kind of calibration which has to be done after each edge change is the cell height alignment to enable the focus of the X-ray beam on the catalyst bed. This height alignment is also employed in the measurement macro and therefore performed automated, but at each edge change.
The higher stability over time of a laboratory X-ray source compared to a synchrotron source is beneficial in terms of required alignments. This higher stability over time allows to perform only the initial calibration before the start of the operando experiments. Concerning the mechanical precision in the laboratory, all mechanics are precise within a few microns while the beam and sample size are within mm. As the mechanical precision of the laboratory-based setup is good enough, it is not necessary to perform any further calibration during the operando experiment.

Each operando experiment consists of an in situ pre-reduction of the catalyst materials and subsequent CO_2 hydrogenation reaction (Figure S3). Depending on the amounts of elements in the catalyst, and therefore, amount of absorption edges, the holding times at each temperature step are adjusted.

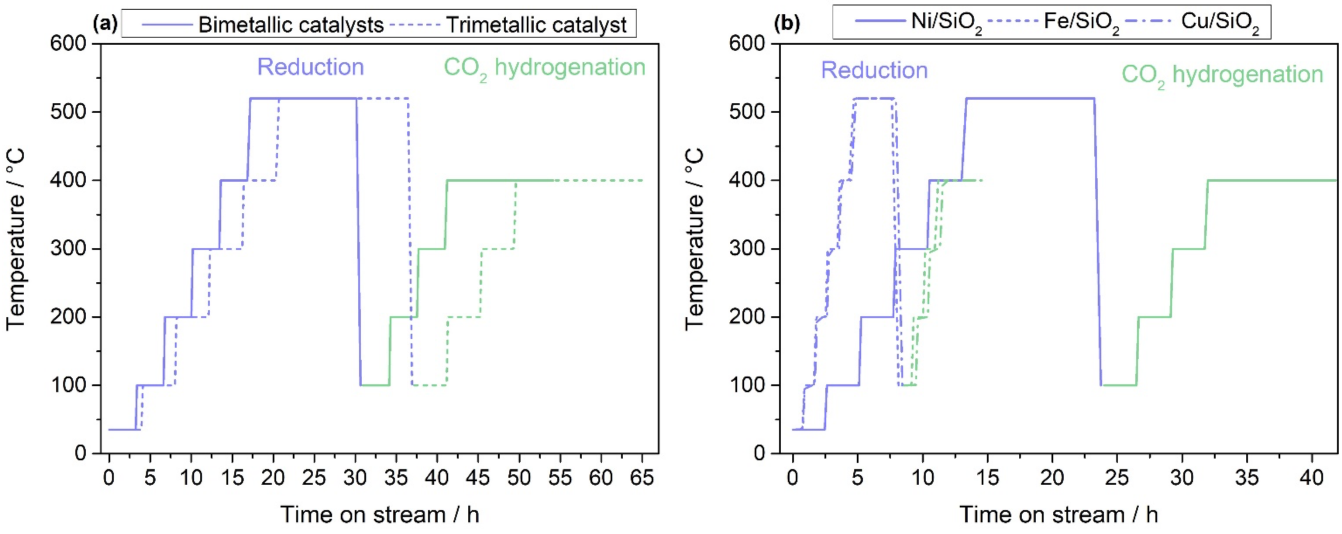

Figure S3. Experimental scheme for the operando laboratory-based XANES experiments of the bi- and trimetallic catalysts (a) and all monometallic counterparts (b). Each operando experiment starts with an in situ pre-reduction of the catalysts (blue) followed by the CO_2 hydrogenation reaction (green).

Thermodynamic Calculations

Thermodynamic calculations of the equilibrium conversions for the Sabatier and reverse water gas shift reactions were performed with the HSC 9.6.1 software in the Gem equilibrium composition mode being based on the Gibbs free energy minimization.

Results and Discussion

Standard Catalyst Characterization

Standard characterization with X-ray diffraction (XRD) and transmission electron microscopy (TEM) does not yield unambiguously conclusive results, especially due to the small particle size of the metal(oxides) on the amorphous SiO_2 support material (Figure S4-S13). Moreover, charging effects of the samples make TEM studies very challenging. Hence, operando laboratory-based XANES constitutes a powerful analytical tool for the characterization of supported multielement metal-based catalysts.

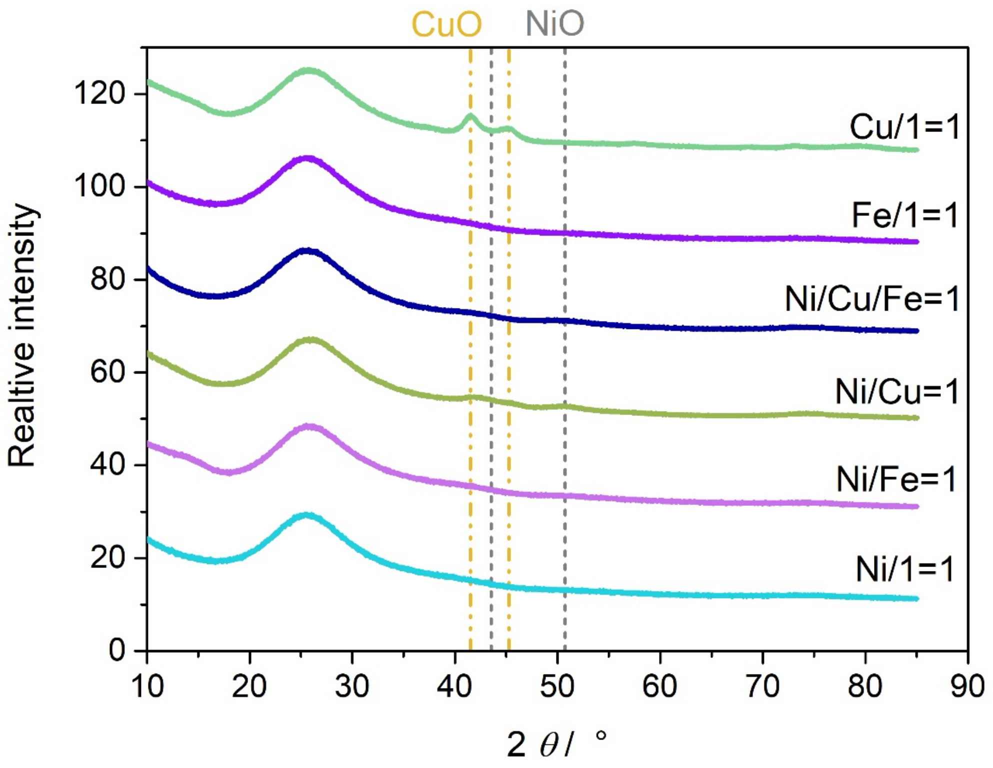

Figure S4. X-ray diffraction (XRD) patterns of all as-prepared catalyst materials under study. The position of the main diffraction peaks of NiO are highlighted as dotted lines (grey) and those of CuO as dash-dotted lines (orange).

For the measurements at the JEOL JEM-2100F microscope, metal nanoparticle sizes were determined in the marked regions (orange lines) of higher contrast in the TEM images. Determining particle size distributions was not feasible due to the low contrast of all catalyst materials under investigation. Nevertheless, based on the TEM images collected (shown in Figures S4-S10), we can conclude that the average metal nanoparticle size for all as-prepared catalyst materials is ~2 nm and there is no indication for a distinct increase in the metal nanoparticle size after reaction. For all catalysts, significant beam-induced effects were visible. Hence, only fast acquisitions with minimal electron-beam dose were applied in order to minimize these detrimental effects. This in turn limited the time to focus and restricted data acquiring at higher magnifications.

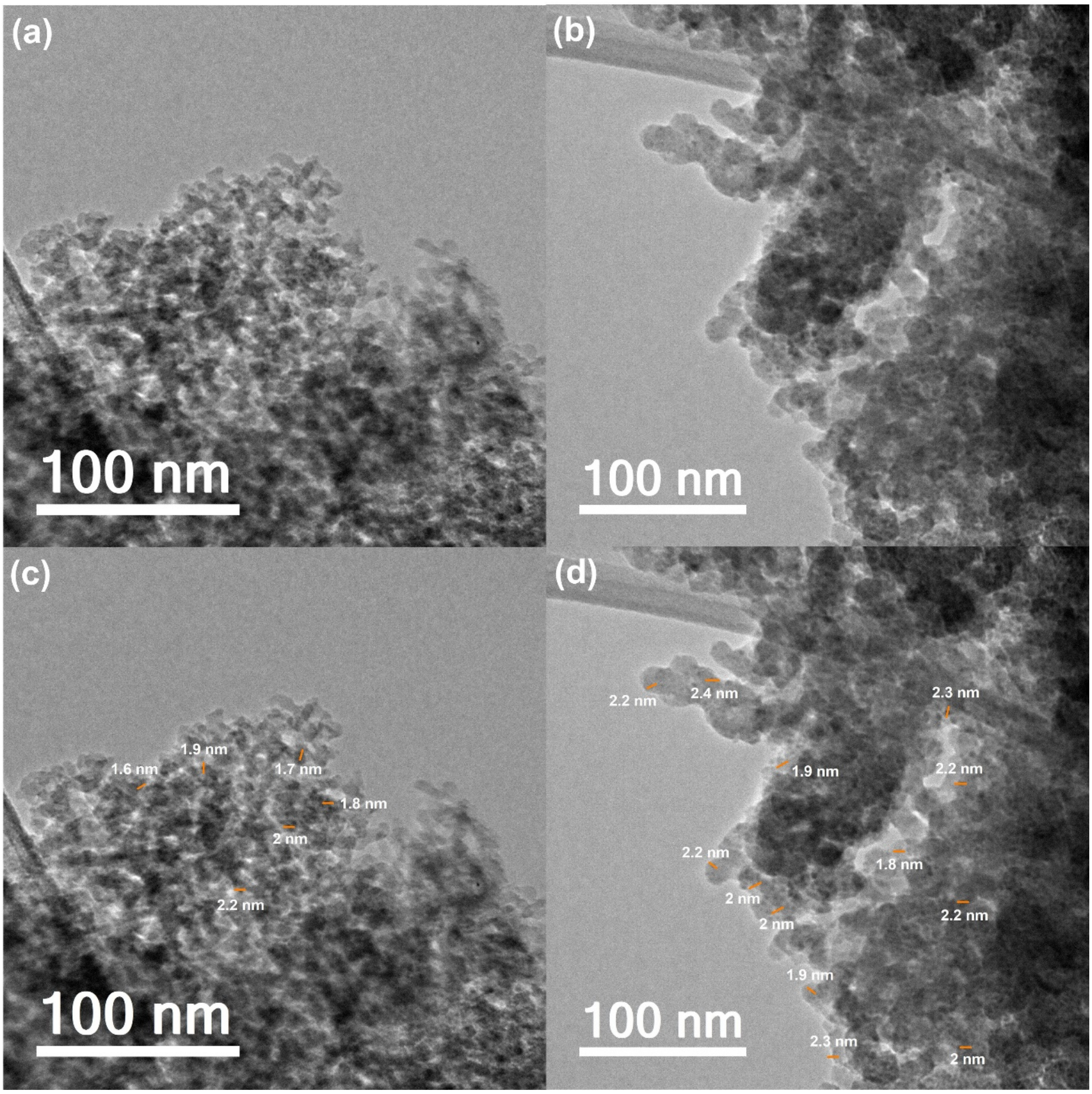

Figure S5. Transmission electron microscopy (TEM) images of the as-prepared (a, c) and spent (b, d) monometallic Ni/SiO_2 catalyst materials. Regions where the particle sizes were determined are depicted as orange lines (c, d).

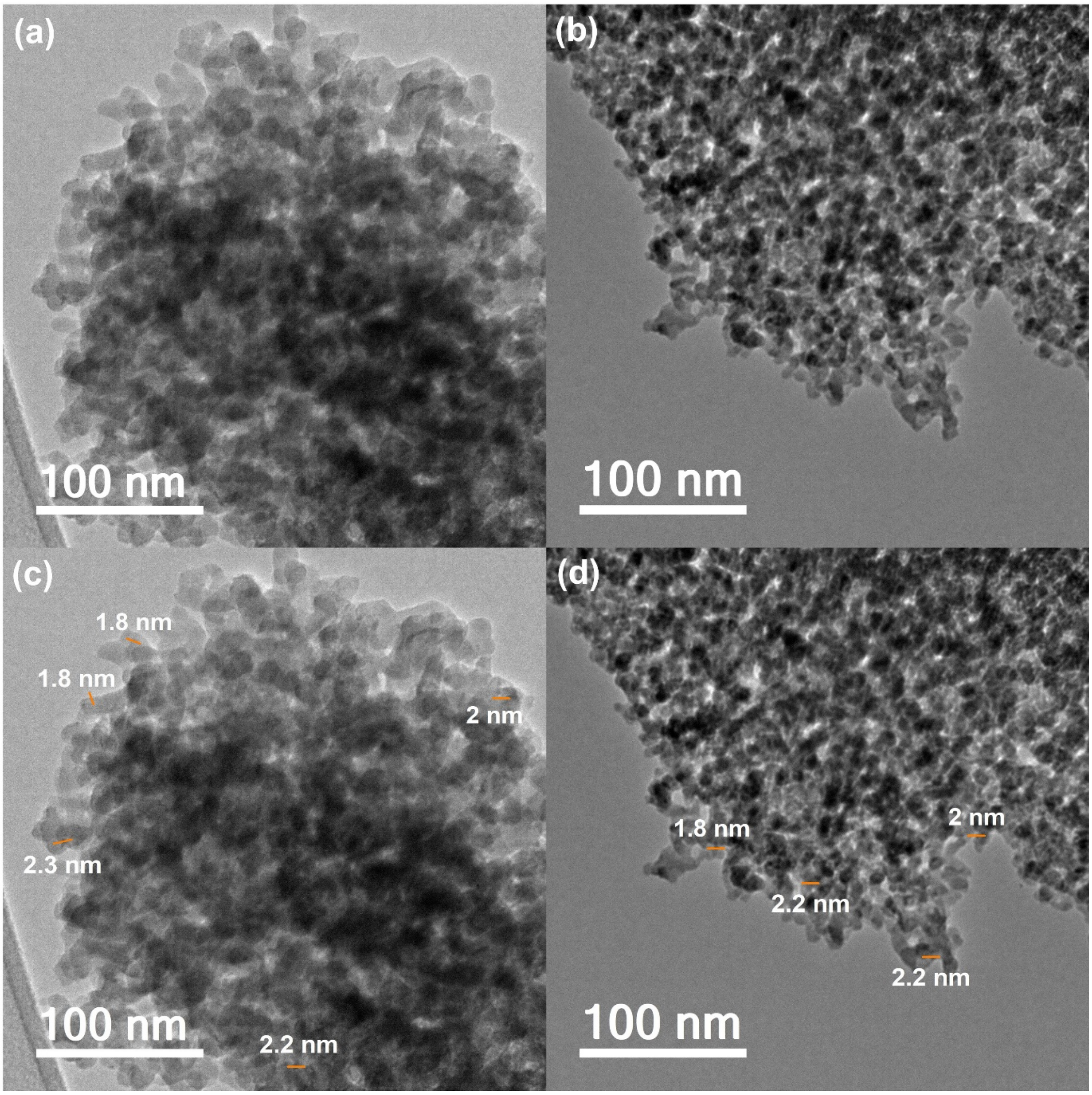

Figure S6. Transmission electron microscopy (TEM) images of the as-prepared (a, c) and spent (b, d) monometallic Fe/SiO_2 catalyst materials. Regions where the particle sizes were determined are depicted as orange lines (c, d).

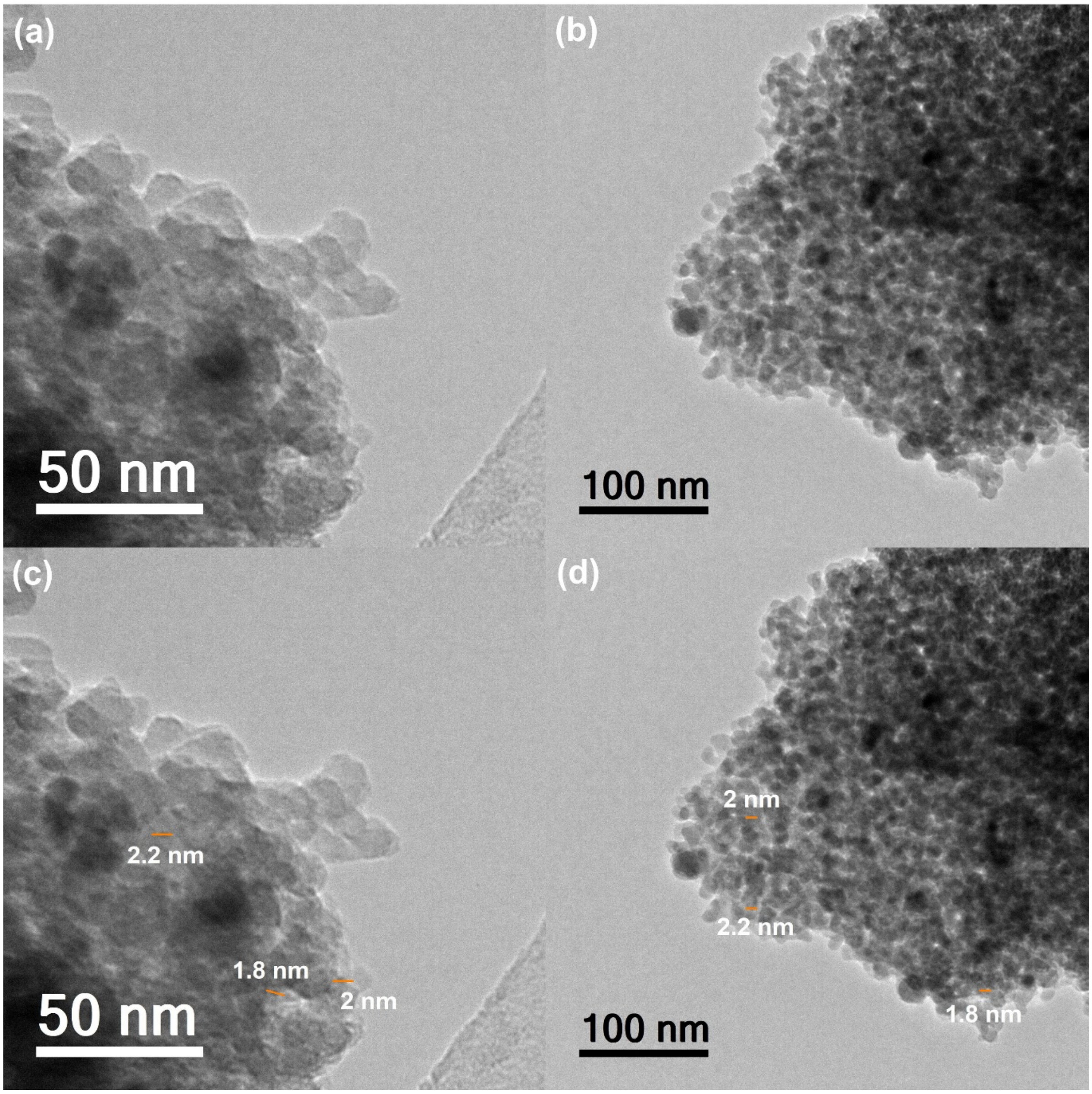

Figure S7. Transmission electron microscopy (TEM) images of the as-prepared (a, c) and spent (b, d) monometallic Cu/SiO_2 catalyst materials. Regions where the particle sizes were determined are depicted as orange lines (c, d).

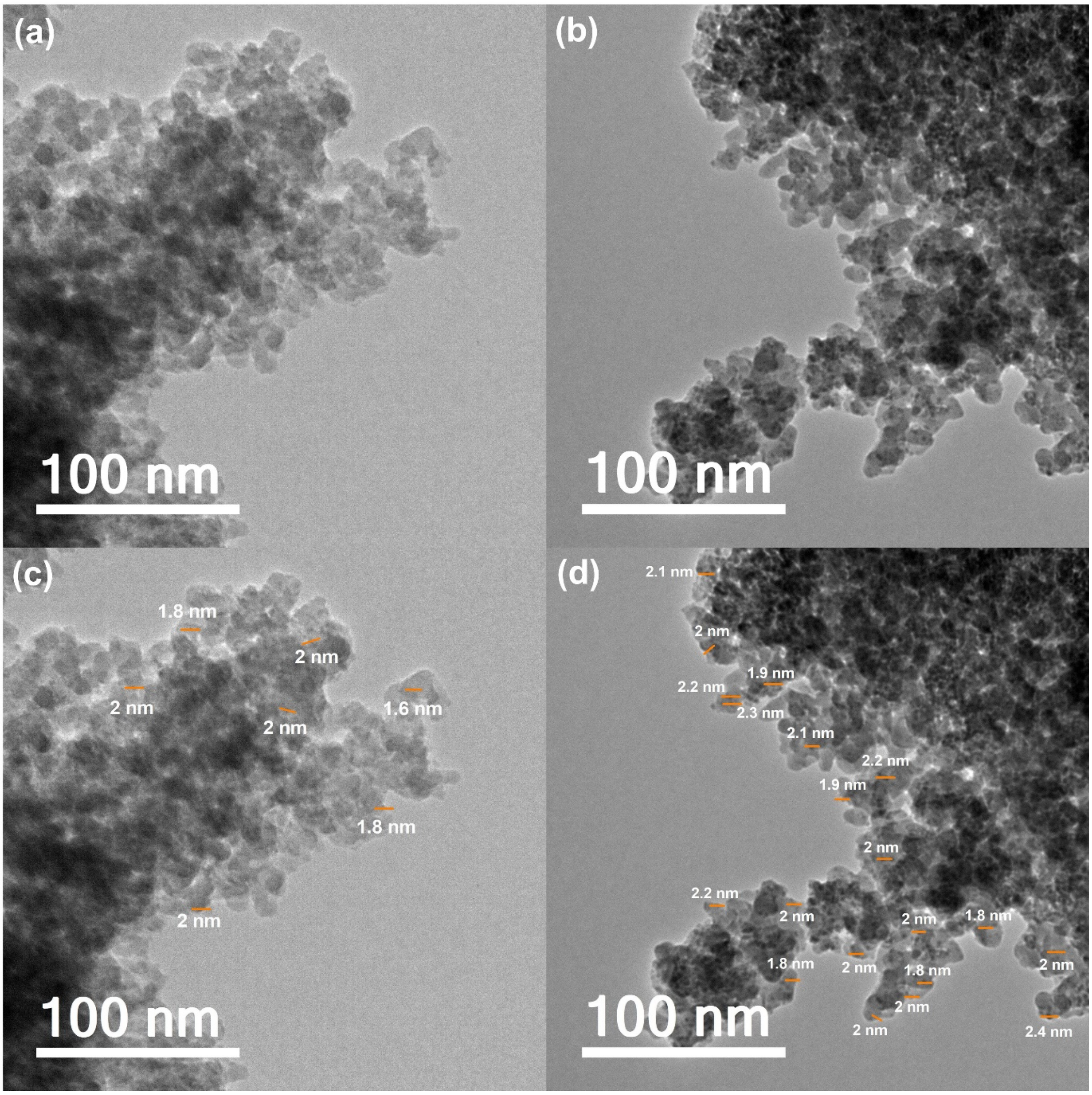

Figure S8. Transmission electron microscopy (TEM) images of the as-prepared (a, c) and spent (b, d) bimetallic Ni-Fe/SiO_2 catalyst materials. Regions where the particle sizes were determined are depicted as orange lines (c, d).

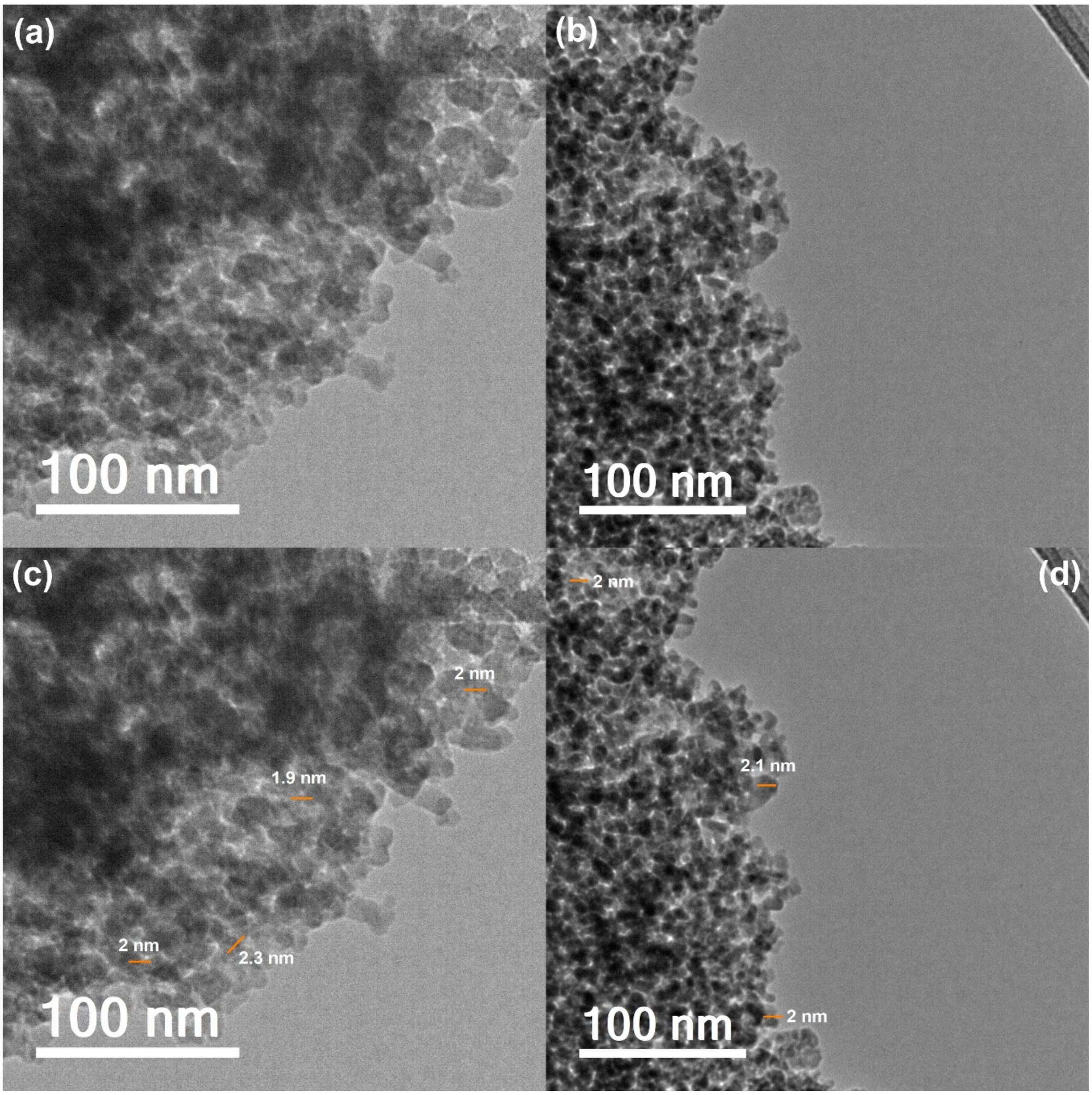

Figure S9. Transmission electron microscopy (TEM) images of the as-prepared (a, c) and spent (b, d) bimetallic Ni-Cu/SiO_2 catalyst materials. Regions where the particle sizes were determined are depicted as orange lines (c, d).

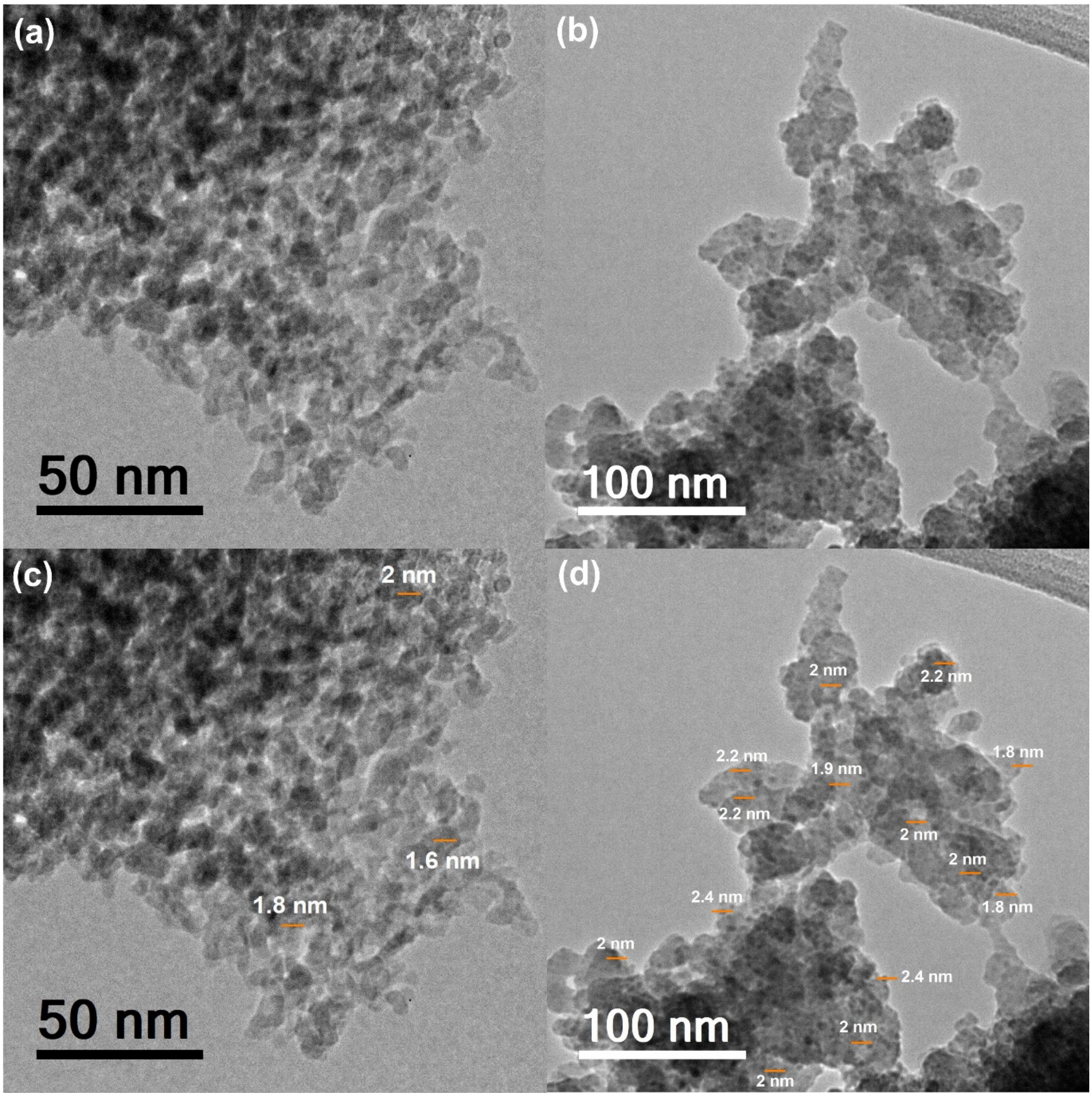

Figure S10. Transmission electron microscopy (TEM) images of the as-prepared (a, c) and spent (b, d) trimetallic Ni-Cu-Fe/SiO_2 catalyst materials. Regions where the particle sizes were determined are depicted as orange lines (c, d).

Particle size distributions were determined from the high-resolution high-angle annular dark-field-scanning transmission electron microscopy (HAADF-STEM) images of all as-prepared and spent catalyst materials (see Figures S11-S16). While the as-prepared catalyst materials consist of metal(oxide) nanoparticles with sizes between ~1 and 2.5 nm, the spent catalyst materials consist of significantly smaller metal nanoparticles ranging from 0.9 to 1.7 nm for the various samples. Based on detailed (S)TEM analysis a distinct increase in particle size due to sintering during reduction and/or CO_2 hydrogenation can be excluded. Only for the monometallic Cu/SiO_2 catalyst a few agglomerates of several small metal nanoparticles (with similar particle sizes) were observed (see Figure S13 a, b).

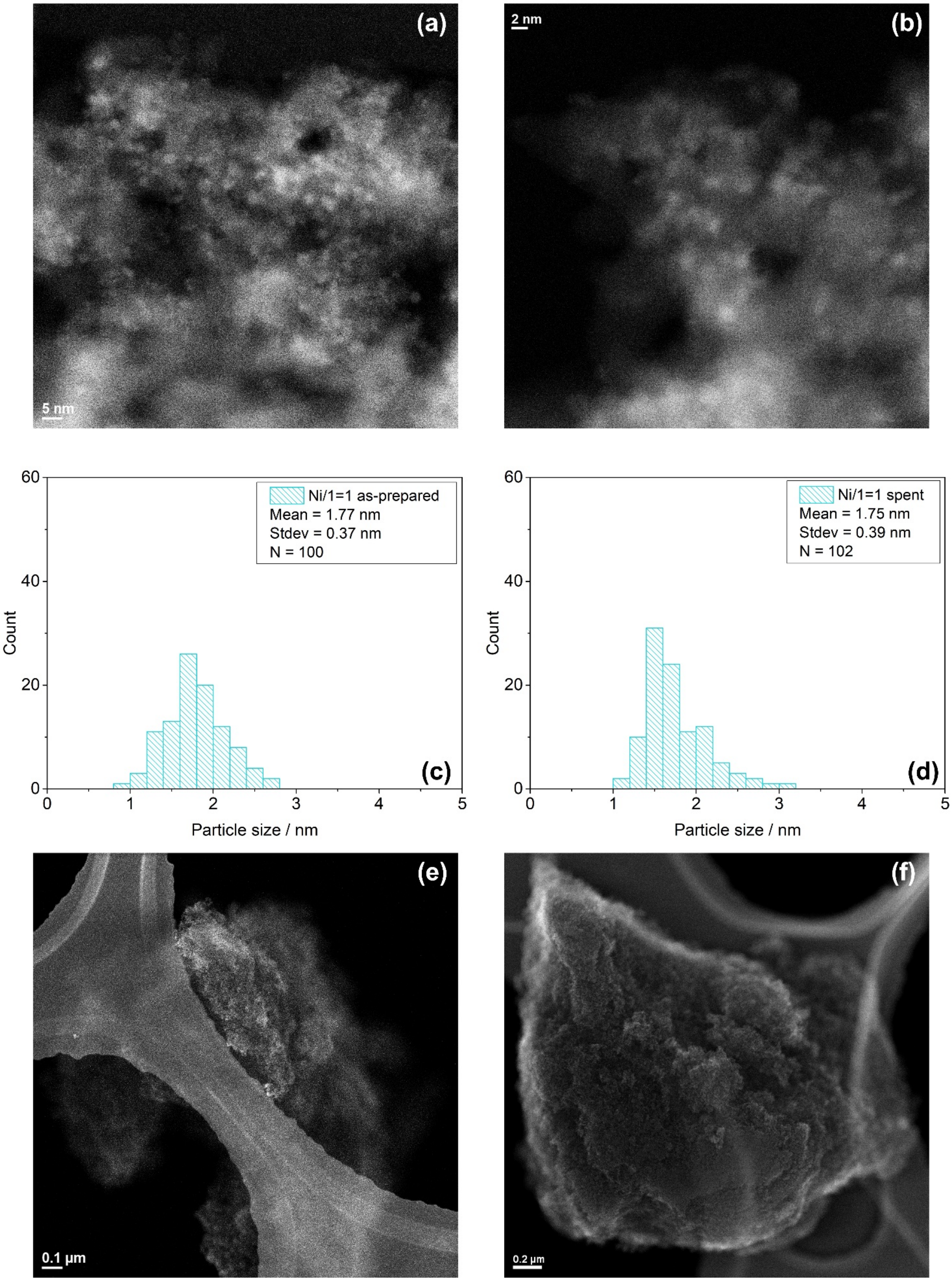

Figure S11. High-angle annular dark-field-scanning transmission electron microscopy (HAADF-STEM) images, therefrom determined metal nanoparticle size distributions, and secondary electron images resulting in information on the catalyst morphology of the as-prepared (a, c, e) and spent (b, d, f) monometallic Ni/SiO_2 catalyst materials.

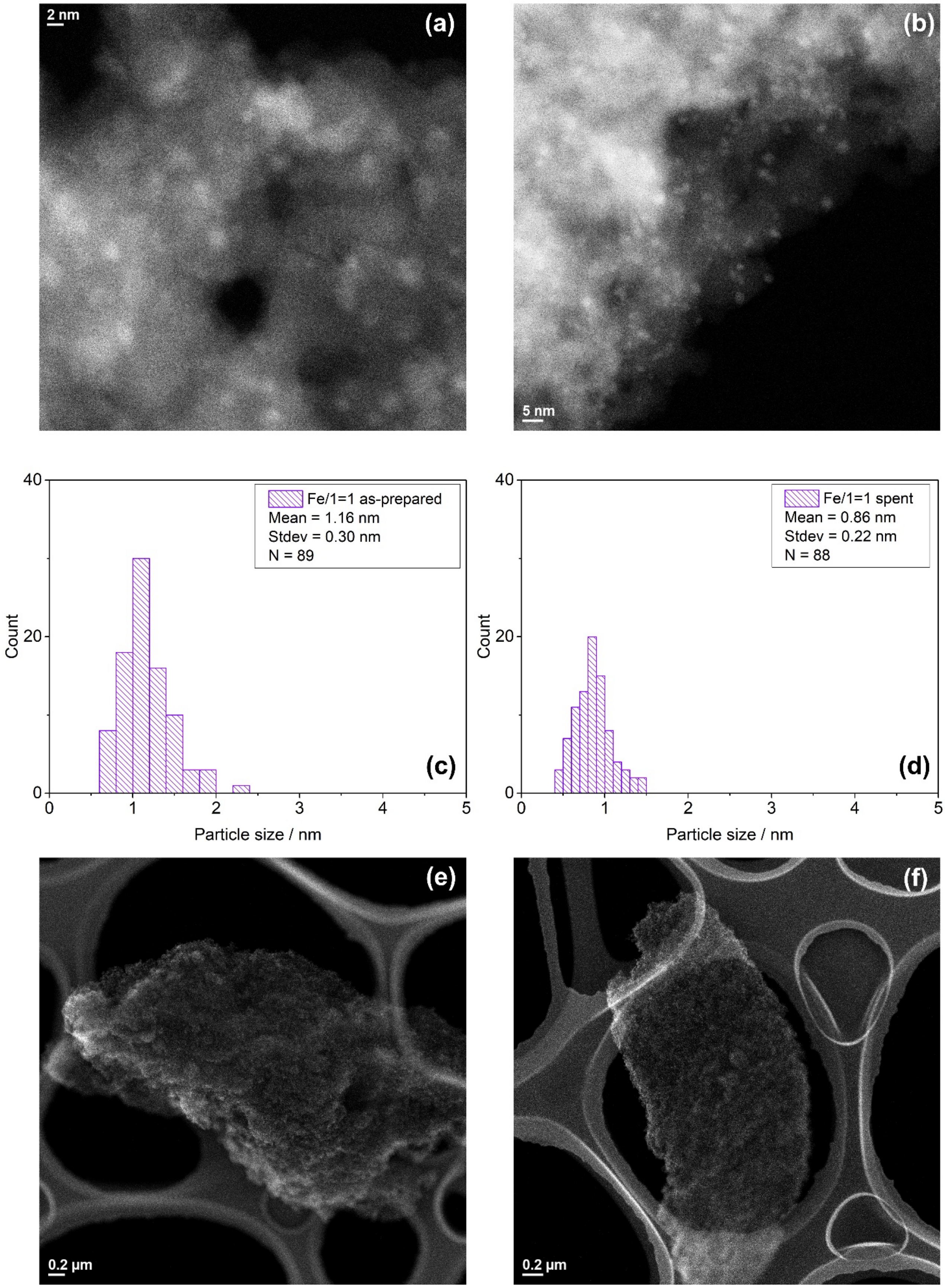

Figure S12. High-angle annular dark-field-scanning transmission electron microscopy (HAADF-STEM) images, therefrom determined metal nanoparticle size distributions, and the secondary electron images resulting in information on the catalyst morphology of the as-prepared (a, c, e) and spent (b, d, f) monometallic Fe/SiO_2 catalyst materials.

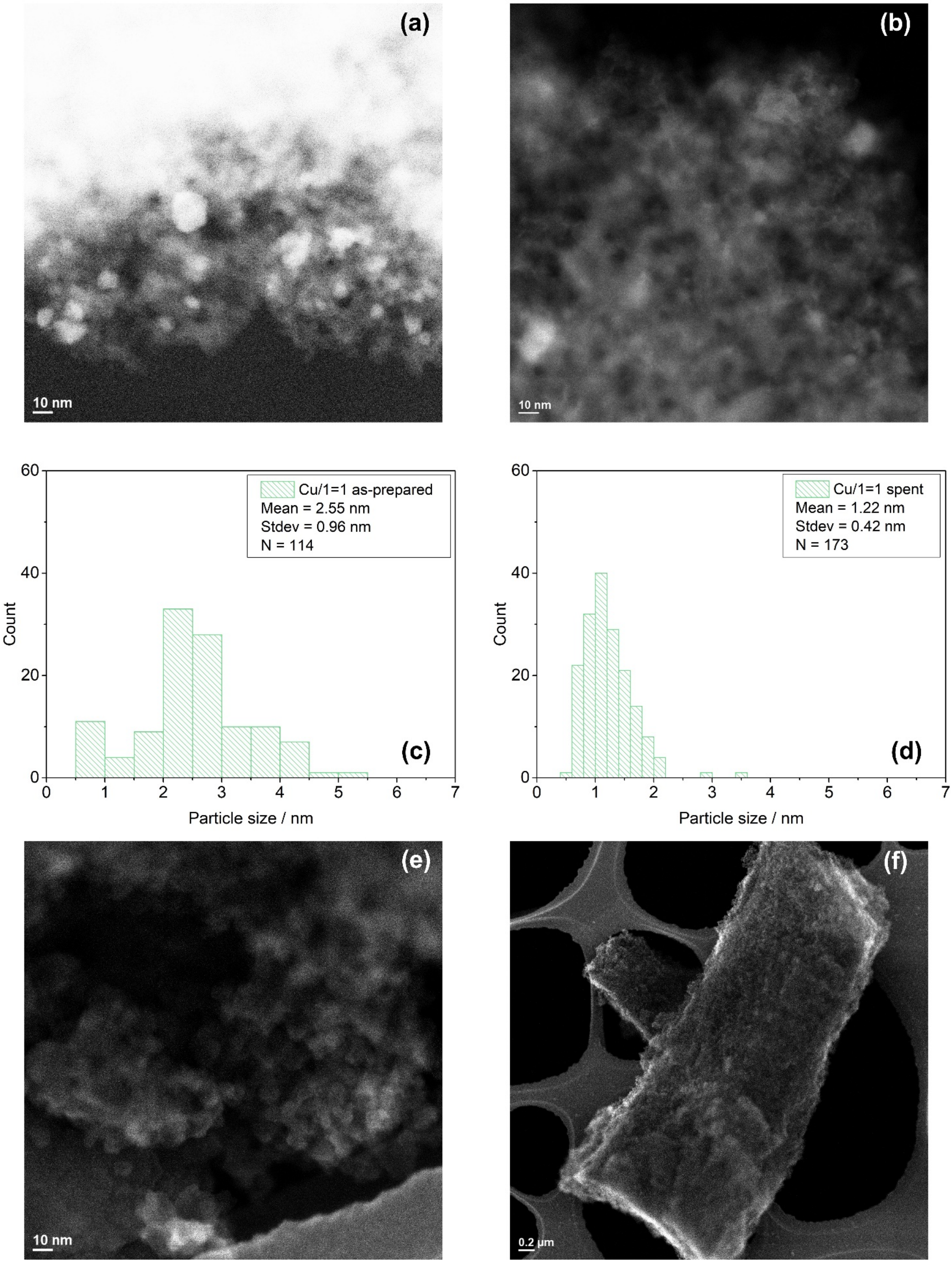

Figure S13. High-angle annular dark-field-scanning transmission electron microscopy (HAADF-STEM) images, therefrom determined metal nanoparticle size distributions, and secondary electron images resulting in information on the catalyst morphology of the as-prepared (a, c, e) and spent (b, d, f) monometallic Cu/SiO_2 catalyst materials.

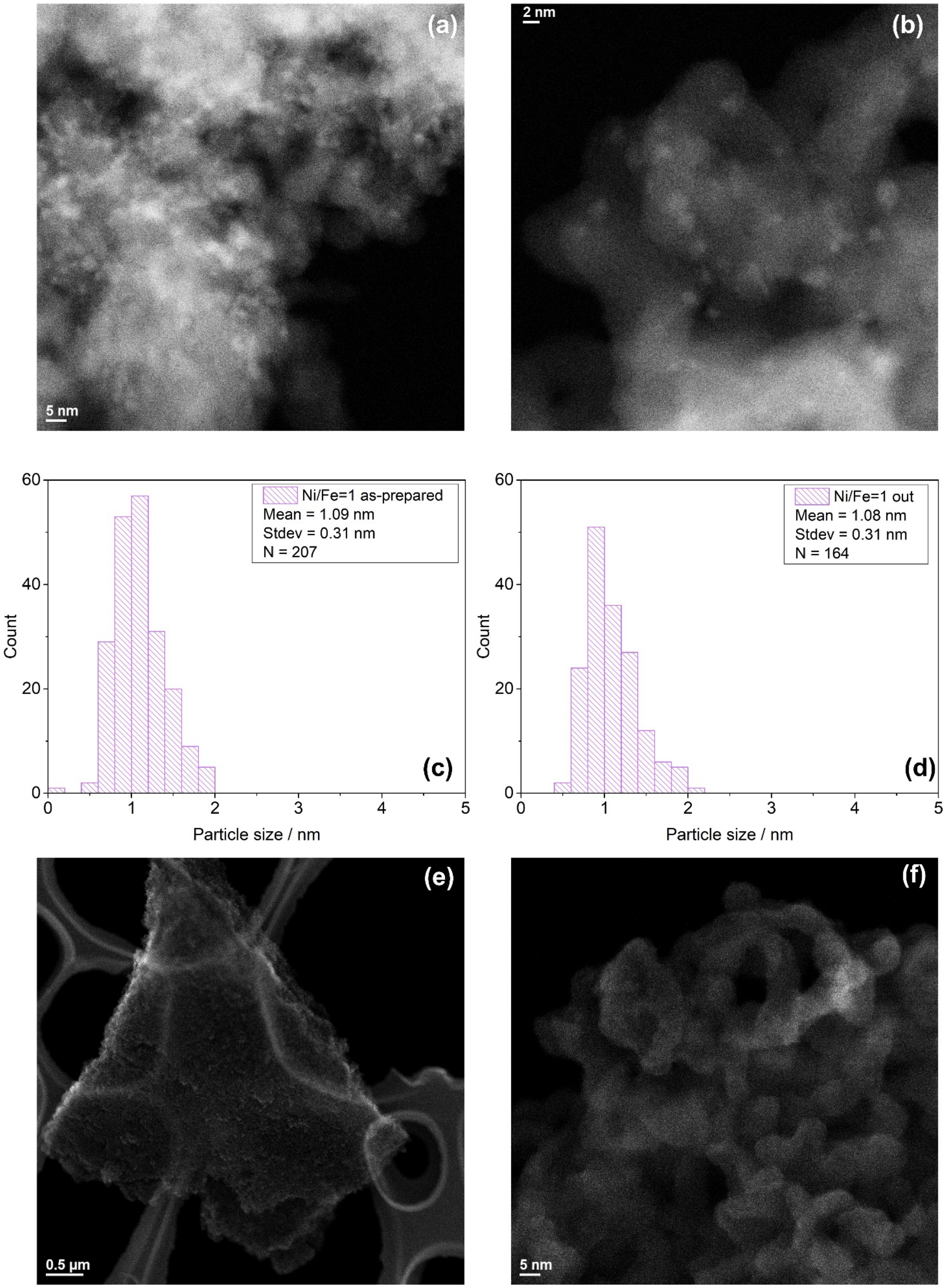

Figure S14. High-angle annular dark-field-scanning transmission electron microscopy (HAADF-STEM) images, therefrom determined metal nanoparticle size distributions, and secondary electron images resulting in information on the catalyst morphology of the as-prepared (a, c, e) and spent (b, d, f) bimetallic Ni-Fe/SiO_2 catalyst materials.

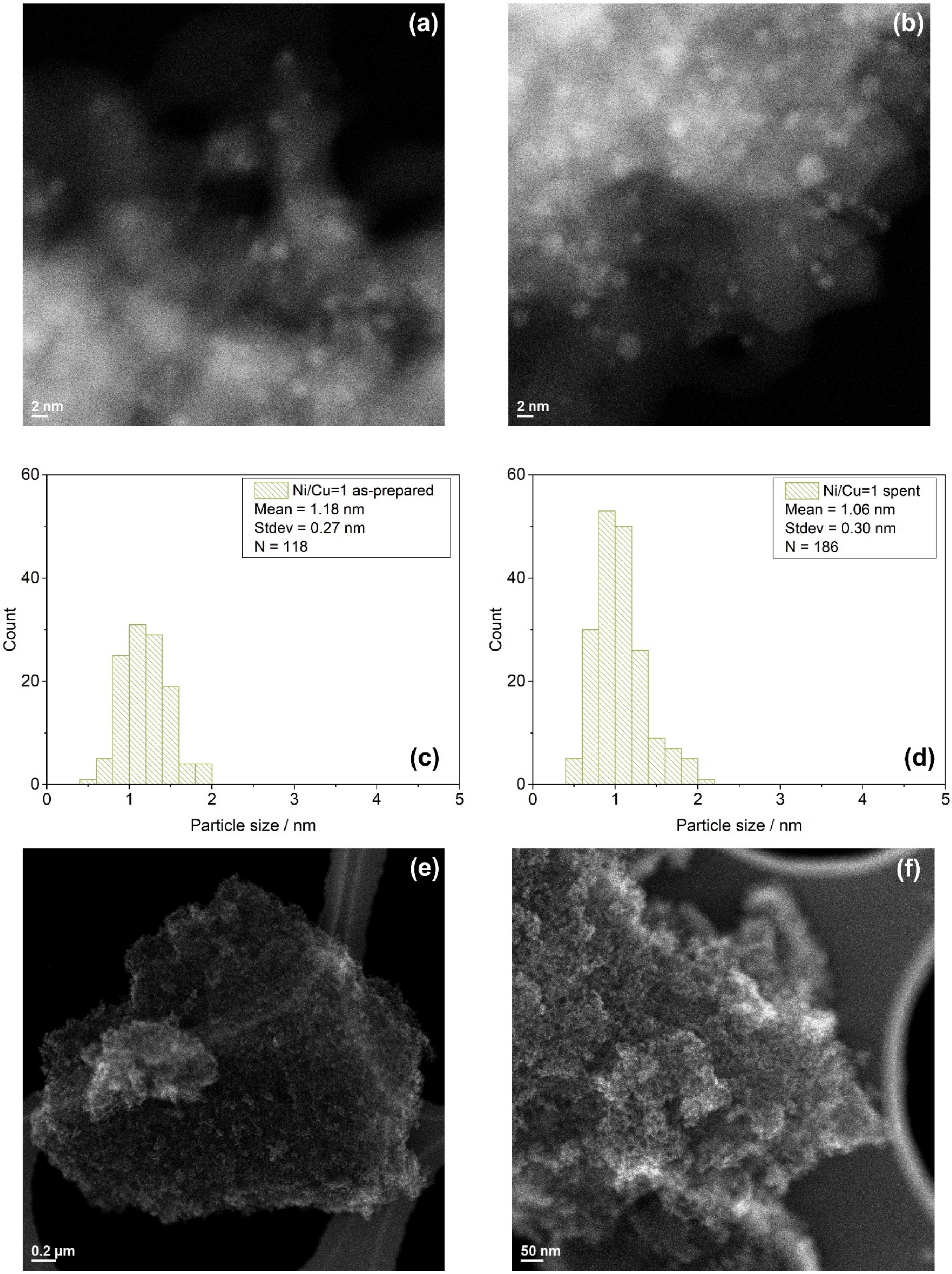

Figure S15. High-angle annular dark-field-scanning transmission electron microscopy (HAADF-STEM) images, therefrom determined metal nanoparticle size distributions, and secondary electron images resulting in information on the catalyst morphology of the as-prepared (a, c, e) and spent (b, d, f) bimetallic Ni-Cu/SiO_2 catalyst materials.

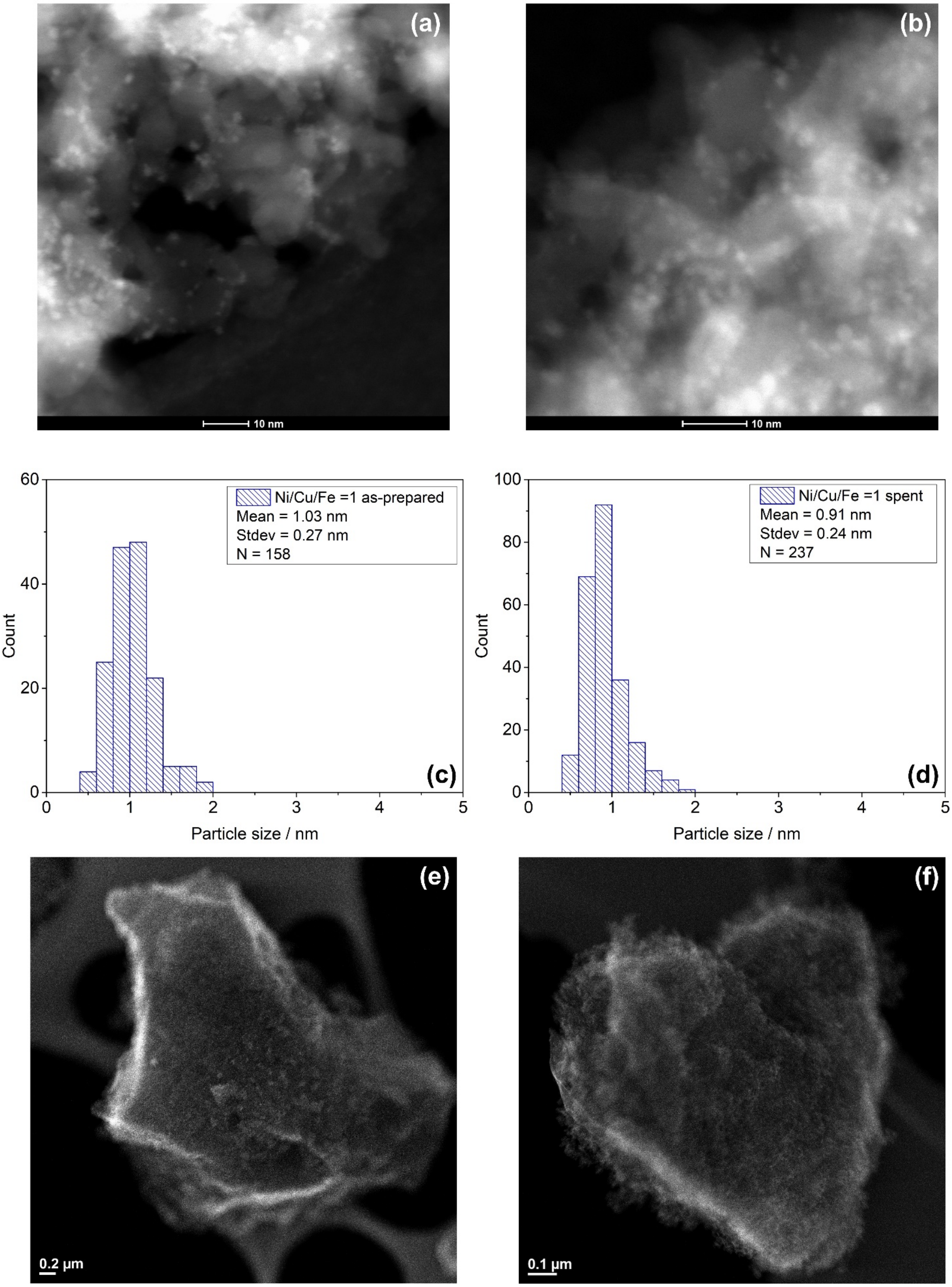

Figure S16. High-angle annular dark-field-scanning transmission electron microscopy (HAADF-STEM) images, therefrom determined metal nanoparticle size distributions, and secondary electron images resulting in information on the catalyst morphology of the as-prepared (a, c, e) and spent (b, d, f) trimetallic Ni-Cu-Fe/SiO_2 catalyst materials.

Catalyst Characterization by Operando Laboratory-based XANES

Possible Systematic Errors of Laboratory-based XAS

Before discussing the resulted laboratory-based XANES, it is important to consider possible systematic errors and evaluate those in view of meaningful data interpretation.

Beam sizes

In general, when performing laboratory-based XAS, the beam size on the sample is comparatively large compared to synchrotron-based experiments. This is due to the fact that the beam size is partially determined by the high-power X-ray tubes, and in part by the SBCA optic which may increase the beam size further especially owing to spherical astigmatism. Toroidally bent crystal analyzers would yield smaller beam spots on the sample than SBCA's, but are currently less common due to a higher price and the fact that the optimal toroid radii depend on the Bragg angle. In the here presented study, the beam size is always smaller or equal compared to the detector size, ensured by a slit system. However, especially when extending the energy range towards the EXAFS region, i.e., going towards lower Bragg angles, the beam size would increase and become significantly larger compared to the detector size. If this is the case, photons will be lost at higher energy. However, even if the beam size reaches the size of the detector, the ratio between I_0 and I would compensate this effect as I_0 is measured in exactly the same configuration. Importantly, in the here presented operando laboratory-based XANES setup, a slit positioned between capillary cell and detector with an opening of 10 mm is employed to keep the horizontal beam dimension at a maximum value of 10 mm, which matches the detector size. Hence, a systematic error due to a large beam size can be excluded for the here presented operando XANES study.

Self-absorption

Self-absorption can occur if the particle size is too large and results in flattened features at the main edge. As the here presented study focuses on small nanoparticles with sizes in the range of 0.7-2.5 nm (Figures S5-S16), an effect of self-absorption is not expected.

Energy calibration

The influence and possible systematic errors induced by a non-proper energy calibration is not specific for laboratory-based studies but holds likewise for synchrotron studies. The energy calibration in the laboratory-based setup is similar to that at a synchrotron facility and performed with standard metal reference foils. The use of SBCA Bragg angles close to 90º renders the need for angular precision to be modest, so the standard mechanical precision of stepper motors, linear stages and goniometers to be highly sufficient. Furthermore, since the heat load on optics can be neglected when using low-brilliance laboratory sources, the presented setup is extremely stable for XAS and systematic errors of the energy calibration can be excluded.

Normalization

In general, systematic errors can be introduced due to possible problems with the normalization of the spectra which holds similarly for both laboratory- and synchrotron-based studies. One main advantage of the normalization when using a laboratory setup is related to the use of a stable laboratory X-ray source, where the incident beam spectrum and intensity (I_0) do not vary with time, leading to a highly stable normalization. All XANES discussed in this manuscript are normalized to this highly stable I_0 spectrum, and resulting spectra are background subtracted and area normalized according to generally accepted XAS data analysis procedures. Systemic errors induced by normalization issues are thus not expected.

Energy resolution

When using a laboratory-based setup, the energy resolution can be lower compared to a synchrotron facility, which holds especially for low Bragg angles. Hence, the direct comparison of laboratory- and synchrotron-based spectra might show deviations. Accordingly, it is important to measure e.g. all reference materials used for LCF with the same setup and exactly the same experimental parameters in order to compensate a possible systematic error due to a lower energy resolution. This procedure has been performed in the here presented operando XANES study and a systematic error induced by a lower energy resolution can therefore be excluded.

Estimation of the Energy Resolution at the Different Absorption Edges

The energy resolution of the here presented laboratory-based XANES setup depends on the Bragg angle, and hence, on the absorption edge. Especially at low Bragg angles, the energy resolution becomes lower compared to a synchrotron facility. The energy resolution is estimated to be 3.2 eV at the Ni K edge (Si(551)), 3.5 eV at the Cu K edge (Si(553)), and 4.6 eV at the Fe K edge (Si(531)). Hence, it is important to measure all reference compounds for e.g. for LCF with the same setup and the same parameters in order to keep the energy resolution for data analysis constant.

Comparison of Metal Foils Measured in the Laboratory-based Setup and at a Synchrotron Facility

A comparison of the Ni (a), Fe (b), and Cu (c) metal foils measured in the laboratory-based operando setup with tabulated metal foils measured at a synchrotron facility[4,5] are depicted in Figure S17. All metal foils are measured after the operando experiment with the same experimental parameters which are used for the operando experiments. Due to the low Bragg angle when measuring at the Fe K edge, the energy resolution is lower compared to corresponding measurements at the Ni and Cu K edge. This in turn results in the expected and observed slight deviation between the Fe foil measured in the laboratory setup and that measured at a synchrotron facility. The use of a Ge(620) instead of the here used Si(531) crystal would improve the energy resolution at the Fe K edge. Alternatively, an improvement of the overall energy resolution could be achieved by slitting the monochromator in the dispersive direction, however, this would at the same time induce a decrease in the count rate. Moreover, the deviation of the laboratory-based and tabulated XANES of the Cu foil could result from a lower quality of the Cu analyzer. In general, it would be possible to achieve a better resolution when using SBCA's with a radius of 1 m instead of the here employed SBCA's with a radius of 0.5 m. However, we opted to use SBCA's with a bending radius of 0.5 m owing to their larger solid angle, resulting in a higher throughput than with SBCA's with 1 m bending radius.

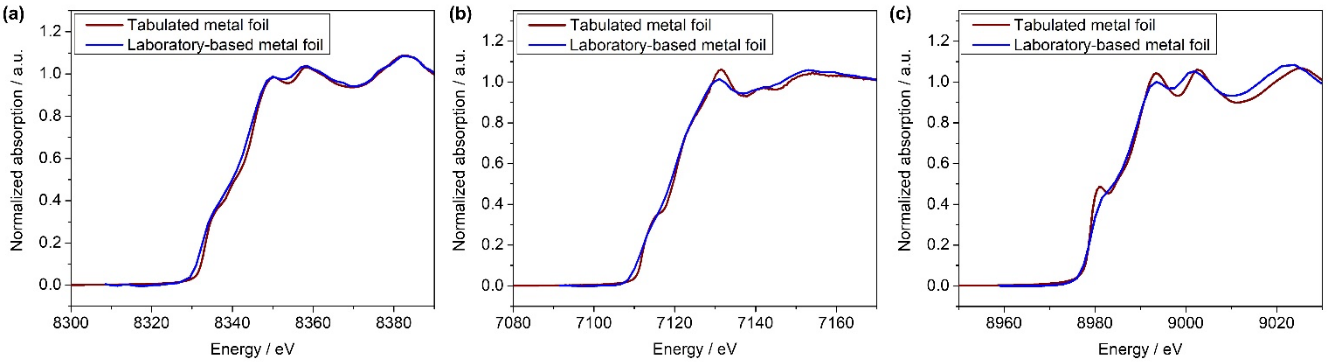

Figure S17. Comparison of the Ni (a), Fe (b), and Cu (c) metal foils measured in the laboratory-based operando setup (blue line) and tabulated ones measured at a synchrotron facility (grey line).[4,5]

Operando XANES Results of the Monometallic Catalysts

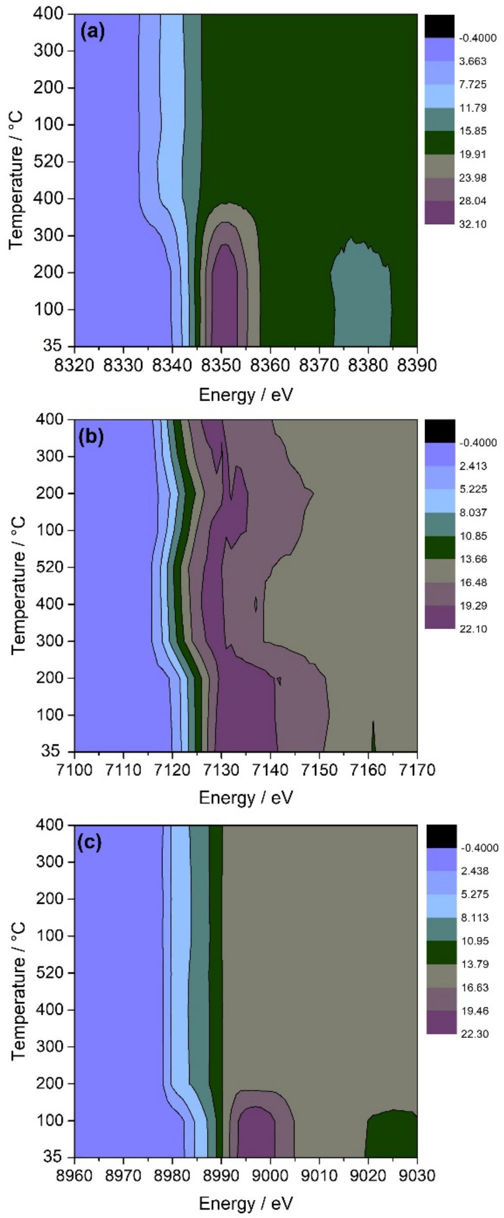

Figure S18. Contour plots of the operando X-ray absorption near-edge structure (XANES) data recorded during reduction (H_2:He = 1:1, 35-520°C, 1 bar) and catalytic CO_2 hydrogenation (H_2:CO_2:He = 4:1:5, 100-400°C, 1 bar) for the monometallic Ni/SiO_2 (a), Fe/SiO_2 (b), and Cu/SiO_2 (c) catalyst materials.

Operando XANES Results of the Multi-Metal Catalyst Materials

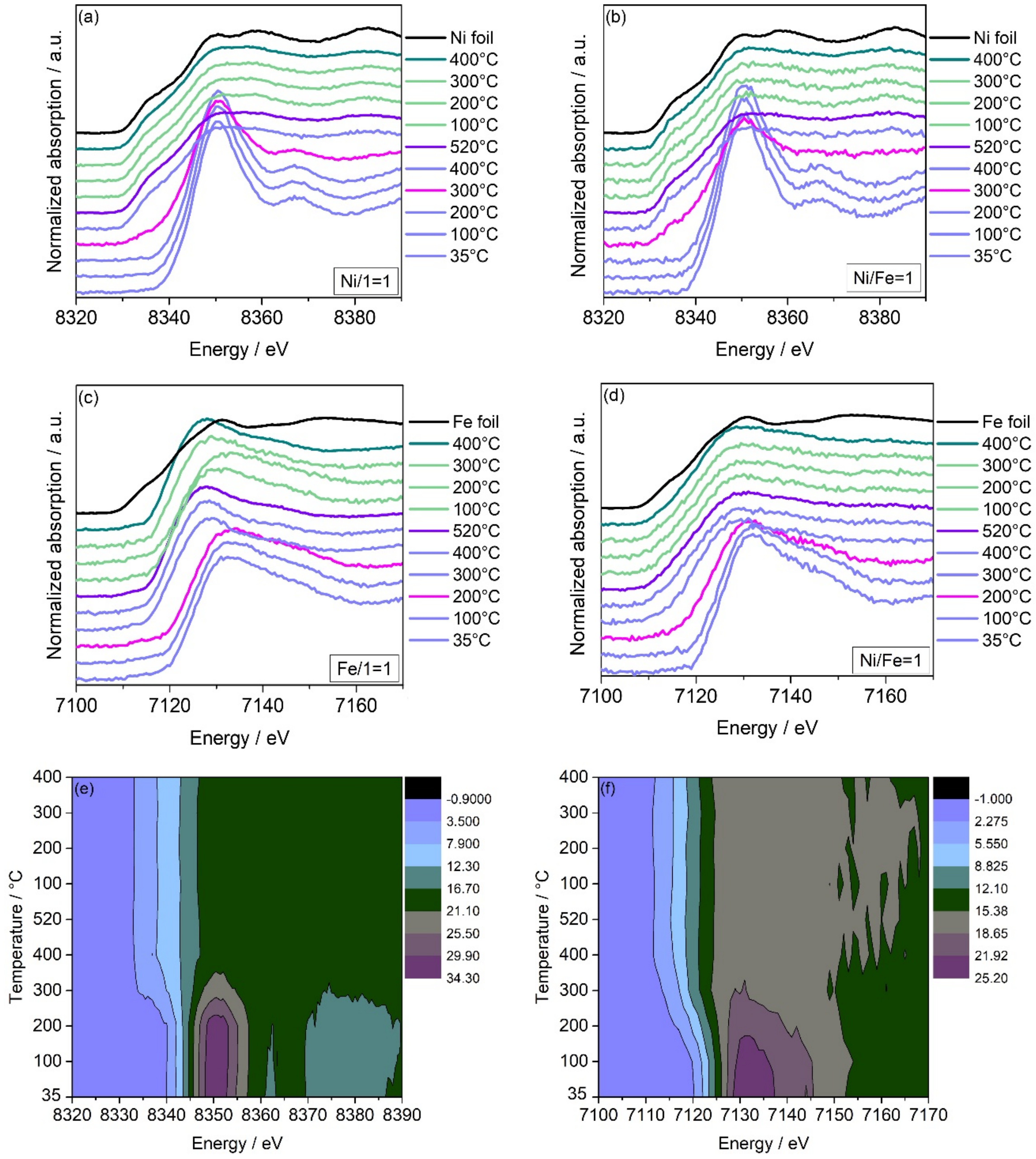

Figure S19. Operando X-ray absorption near-edge structure (XANES) data recorded during reduction (blue/magenta, H_2:He = 1:1, 35-520°C, 1 bar) and catalytic CO_2 hydrogenation (green, H_2:CO_2:He = 4:1:5, 100-400°C, 1 bar) for the bimetallic Ni-Fe/SiO_2 (Ni/Fe=1) and corresponding monometallic catalysts at the Ni (a, b) and Fe (c, d) K edge together with corresponding contour plots for the bimetallic Ni-Fe/SiO_2 catalyst at the Ni (e) and Fe (f) K edge. Significant differences in the reducibility of the bimetallic compared to corresponding monometallic catalysts are highlighted in magenta (a-d).

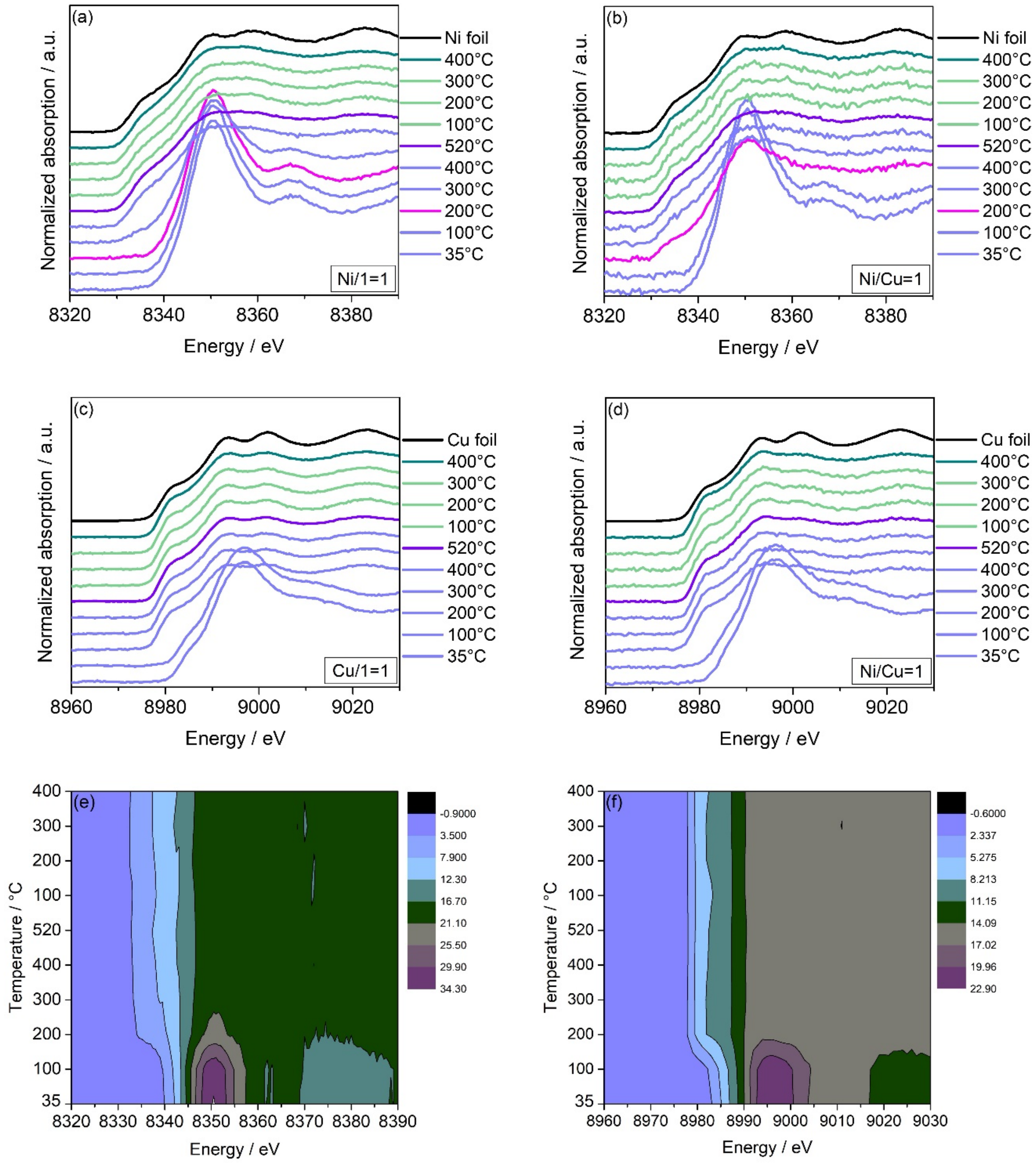

Figure S20. Operando X-ray absorption near-edge structure (XANES) data recorded during reduction (blue/magenta, H_2:He = 1:1, 35-520°C, 1 bar) and catalytic CO_2 hydrogenation (green, H_2:CO_2:He = 4:1:5, 100-400°C, 1 bar) for the bimetallic Ni-Cu/SiO_2 (Ni/Cu=1) and corresponding monometallic catalysts at the Ni (a, b) and Cu (c, d) K edge together with corresponding contour plots for the bimetallic catalyst at the Ni (e) and Cu (f) K edge. Significant differences in the reducibility of the bimetallic compared to corresponding monometallic catalysts are highlighted in magenta (a, b).

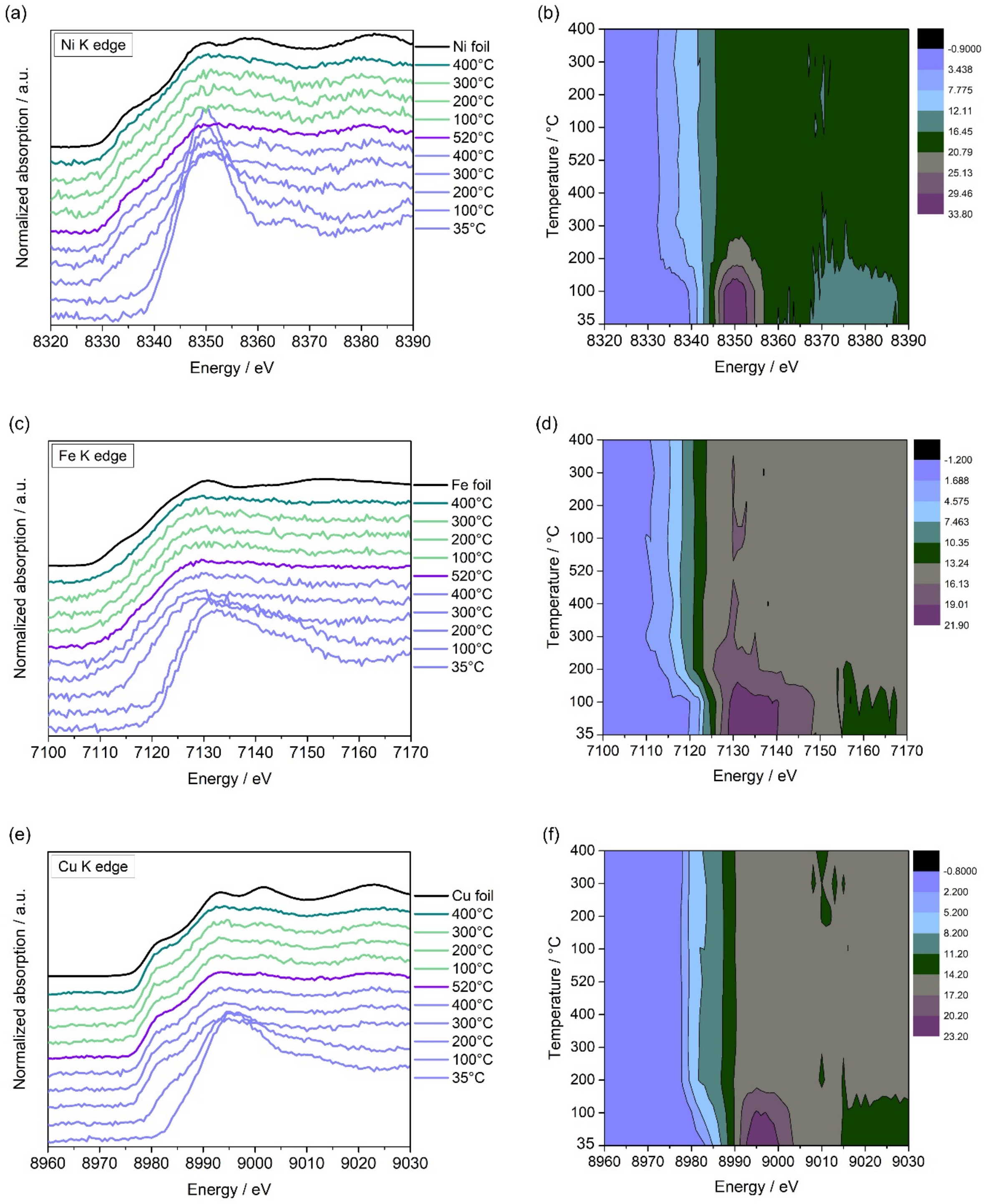

Figure 21. Operando X-ray absorption near-edge structure (XANES) recorded during reduction (blue, H_2:He = 1:1, 35-520°C, 1 bar) and CO_2 hydrogenation (green, H_2:CO_2:He = 4:1:5, 100-400°C, 1 bar,) for the trimetallic Ni-Cu-Fe/SiO_2 catalyst at the Ni (a), Fe (c), and Cu (e) K edge and corresponding contour plots (b, d, f).

Average Oxidation States of the Species of the Supported Catalyst Materials

The position of the first maximum of the first derivative of the XANES can be used for determining the average oxidation state of the species of the supported catalyst materials. It is important to note that this approach yields only average oxidation states and cannot distinguish variations in oxidation state based on differences in particle size – it reports the average oxidation state based on the particle size distribution. Moreover, results based on the comparison of the oxidation states of metal(oxide) reference compounds and supported catalysts have to be interpreted with caution and the particle size difference between the supported nanoparticles and the reference compounds has to be taken into account. Considering these points, the position of the first maximum of the first derivative of the XANES

as function of temperature during reaction, i.e., first during reduction and subsequently during CO_2 hydrogenation, reveals distinctly pronounced trends which are in accordance with the XANES at the Ni, Fe, and Cu K edge (Figure S22 a-c).

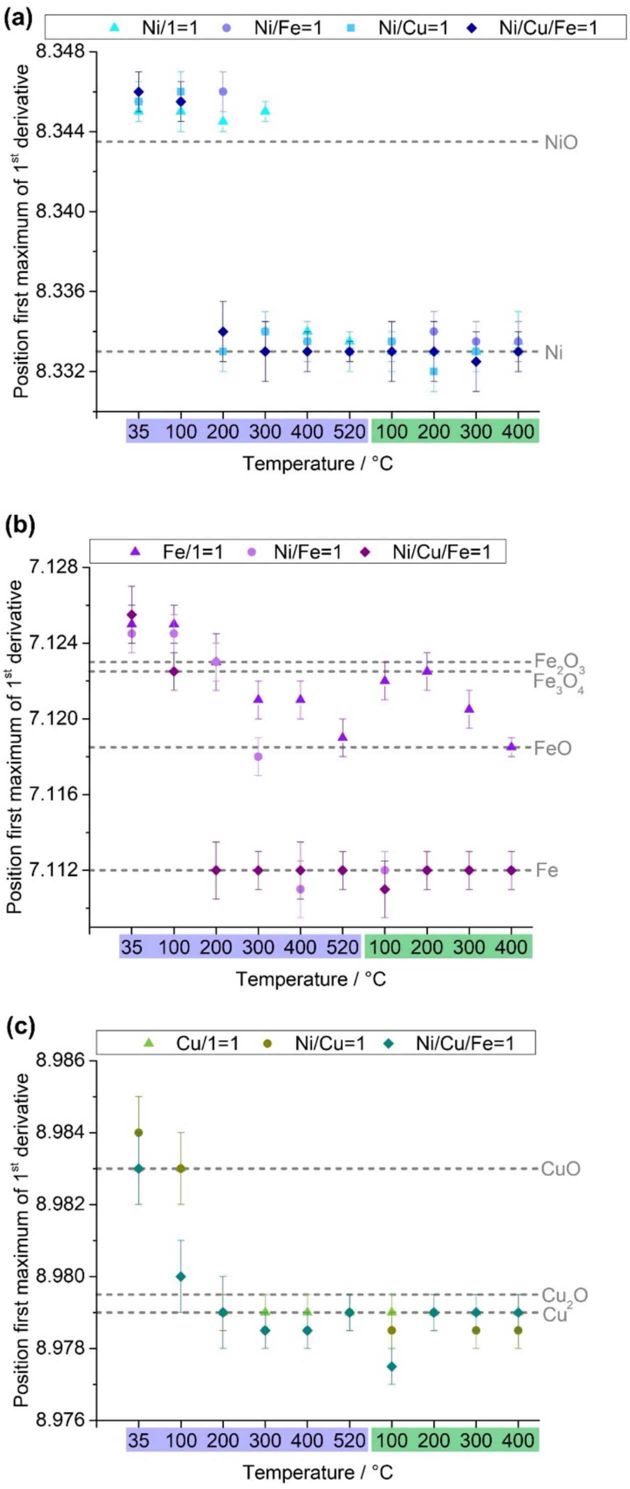

Figure S22. Position of the first maximum of the first derivative of the X-ray absorption near-edge structure (XANES) during reduction (blue temperatures on x-axis, H_2:He = 1:1, 35-520°C, 1 bar) and catalytic CO_2 hydrogenation (green temperatures on x-axis, H_2:CO_2:He = 4:1:5, 100-400°C, 1 bar, GHSV = 4.1 10^4 h^{-1}) at the Ni (a), Fe (b), and Cu (c) K edge for the tri-, bi-, and corresponding monometallic catalyst materials. Errors are determined according to the procedure explained in the Catalyst Characterization of the SI.

For the nickel species, the initial state can be identified as Ni(II) (Figure S22 a), being in accordance with the expected fully oxidized nickel species resulting after calcination. For the monometallic Ni/SiO_2 catalyst, the reduction of the Ni(II) towards Ni(0) species takes place at 400°C. The synergistic effect when forming a bimetallic Ni-Fe and Ni-Cu system is reflected in the facilitated reducibility of Ni(II) towards Ni(0) species (Figure S22 a). When adding copper instead of iron, this effect is more pronounced. Interestingly, within error limits, the reducibility of the initial Cu(II) species towards Cu(I) and Cu(0) species is not affected by the synergistic effect between nickel and copper in the bimetallic catalyst while it is for the trimetallic catalyst (Figure S22 c). Based on the error limits for the position of the first maximum of the first derivative of the XANES, a differentiation between Cu(I) and Cu(0) species cannot be made

unambiguously. The same applies for the differentiation between Fe(III) and Fe(II,III) species. While the iron species of the monometallic Fe/SiO_2 catalyst are not fully reduced, but remain in the oxidation state +II, the synergistic effect between iron and nickel facilitates the reducibility of iron, yielding Fe(0) species (Figure S22 b). These trends in the position of the first maximum of the first derivative of the XANES corroborate the conclusions drawn from the operando XANES alone (Figures S18-21).

Degree of Re-oxidation during CO_2 Hydrogenation Conditions

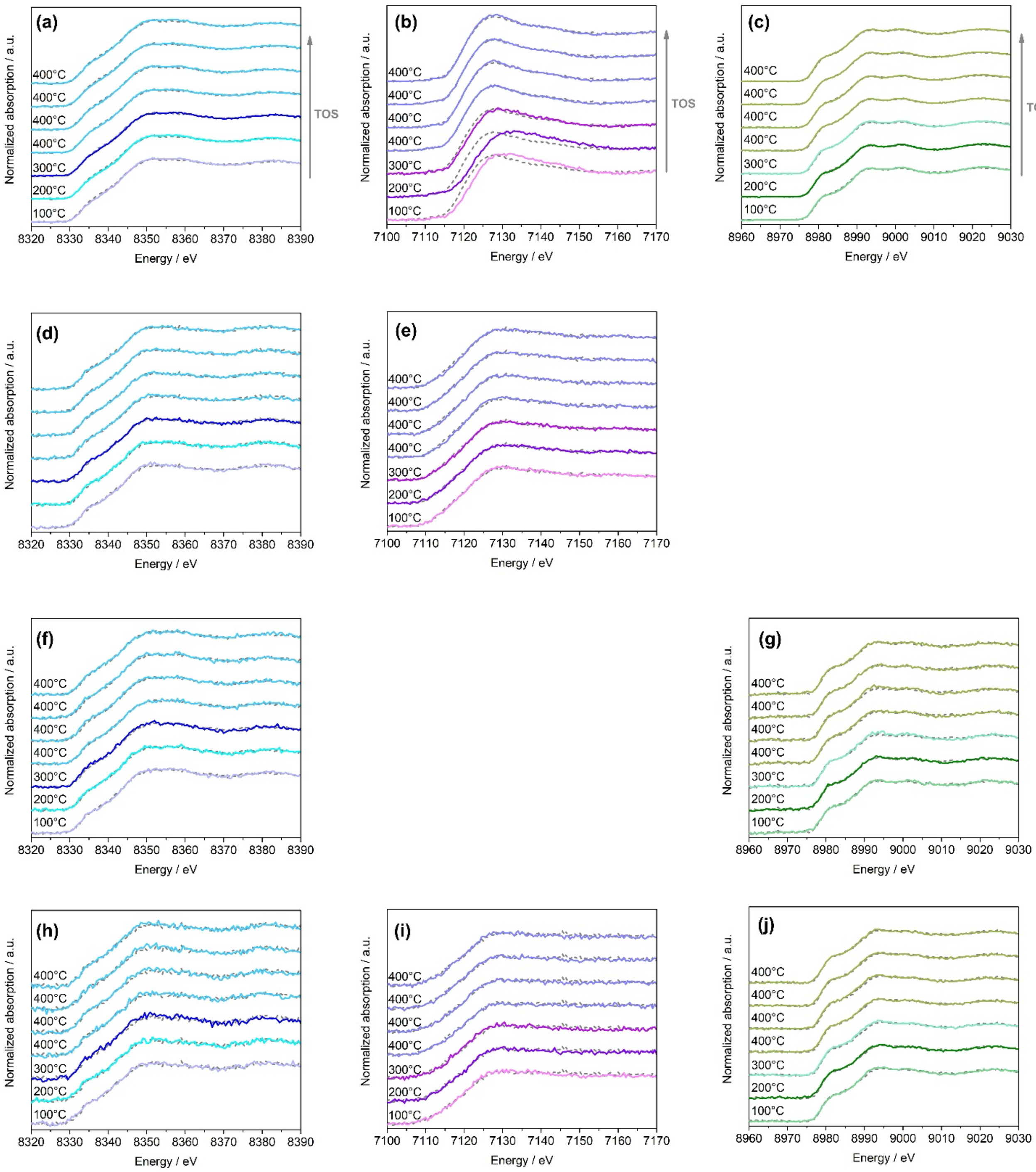

Figure S23. Comparison of the X-ray absorption near-edge structure (XANES) data at the Ni (a, d, f, h), Fe (b, e, i), and Cu (c, g, j) K edge for the mono- (a-c), bi- (d-g), and trimetallic (h-j) catalyst materials of the reduced state at 520°C (grey line, H_2:He = 1:1, 1 bar) and those during catalytic CO_2 hydrogenation at varying temperature (H_2:CO_2:He = 4:1:5, 100-400°C, 1 bar) as indication for the degree of re-oxidation.

Based on the comparison of the XANES of the final reduced state at 520°C during reduction and those during catalytic CO_2 hydrogenation for the multi-metal catalyst materials and corresponding monometallic counterparts, it is evident that neither for the trimetallic nor for the Ni-Fe and Ni-Cu bimetallic catalysts a distinct evolution with time on stream during CO_2 hydrogenation can be revealed. Hence, it is legitimate to average the spectra at 400°C during CO_2 hydrogenation to increase the S/N ratio. This holds likewise for all monometallic catalysts as the observed re-oxidation of the Fe/SiO_2 catalyst proceeds only at lower temperature.

Linear Combination Fitting

Table S1. Results from linear combination fitting (LCF) of the X-ray absorption near-edge structure (XANES) data obtained at the Ni K edge at 400°C during catalytic CO_2 hydrogenation for various catalyst materials.

Sample	R-factor	Weight (NiO)	Weight (Ni^0)
Monometallic Ni/SiO_2 (Ni/1=1)	0.0036	0.060±0.020	0.940±0.020
Bimetallic Ni-Fe/SiO_2 (Ni/Fe=1)	0.0023	0.032±0.016	0.968±0.016
Bimetallic Ni-Cu/SiO_2 (Ni/Cu=1)	0.0074	0.074±0.029	0.926±0.029
Trimetallic Ni-Cu-Fe/SiO_2 (Ni/Cu/Fe=1)	0.0113	0.057±0.034	0.943±0.034

Table S2. Results from linear combination fitting (LCF) of the X-ray absorption near-edge structure (XANES) data obtained at the Fe K edge at 400°C during catalytic CO_2 hydrogenation for various catalyst materials.

Sample	R-factor	Weight (Fe_3O_4)	Weight (FeO)	Weight (Fe^0)
Monometallic Fe/SiO_2 (Fe/1=1)	0.0629	0.252±0.092	0.748±0.092	-
Bimetallic Ni-Fe/SiO_2 (Ni/Fe=1)	0.0066	0.130±0.092	0.495±0.047	0.375±0.047
Trimetallic Ni-Cu-Fe/SiO_2 (Ni/Cu/Fe=1)	0.0049	0.053±0.083	0.350±0.039	0.597±0.039

Table S3. Results from linear combination fitting (LCF) of the X-ray absorption near-edge structure (XANES) data obtained at the Cu K edge at 400°C during catalytic CO_2 hydrogenation for various catalyst materials.

Sample	R-factor	Weight (CuO)	Weight (Cu_2O)	Weight (Cu^0)
Monometallic Cu/SiO_2 (Cu/1=1)	0.00075	-	0.321±0.022	0.679±0.022
Bimetallic Ni-Cu/SiO_2 (Ni/Cu=1)	0.0016	0.015±0.020	0.543±0.068	0.442±0.040
Trimetallic Ni-Cu-Fe/SiO_2 (Ni/Cu/Fe=1)	0.0040	0.048±0.032	0.606±0.052	0.347±0.094

LCF is performed by using metal oxide reference compounds and corresponding metal foils, which are measured with the laboratory-based setup and depicted in Figure S24.

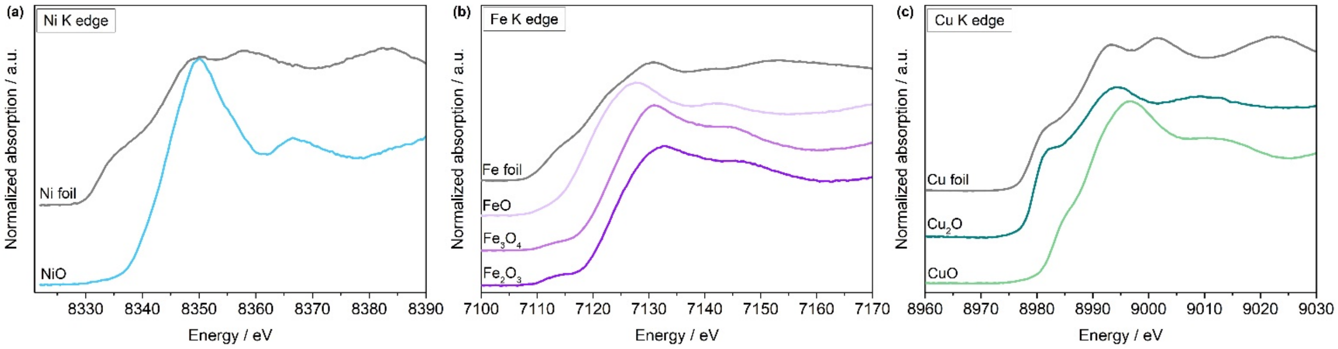

Figure S24. X-ray absorption near-edge structure (XANES) of all considered reference compounds for the linear combination fitting (LCF) of the catalyst materials at the Ni (a), Fe (b), and Cu (c) K edge.

It is evident from Figure S25, that there are some deviations between the experimental and fitted XANES, i.e., the fits show a more pronounced characteristic shape after the absorption edge while the experimental XANES show strongly broadened or even missing features after the absorption edge. This observation is most distinctly pronounced for the Ni and Fe K edge XANES. The broadening or missing of characteristic features can be explained with the small particle size (Figure S5-S16) of the investigated catalyst materials compared to the bulk oxide reference compounds.

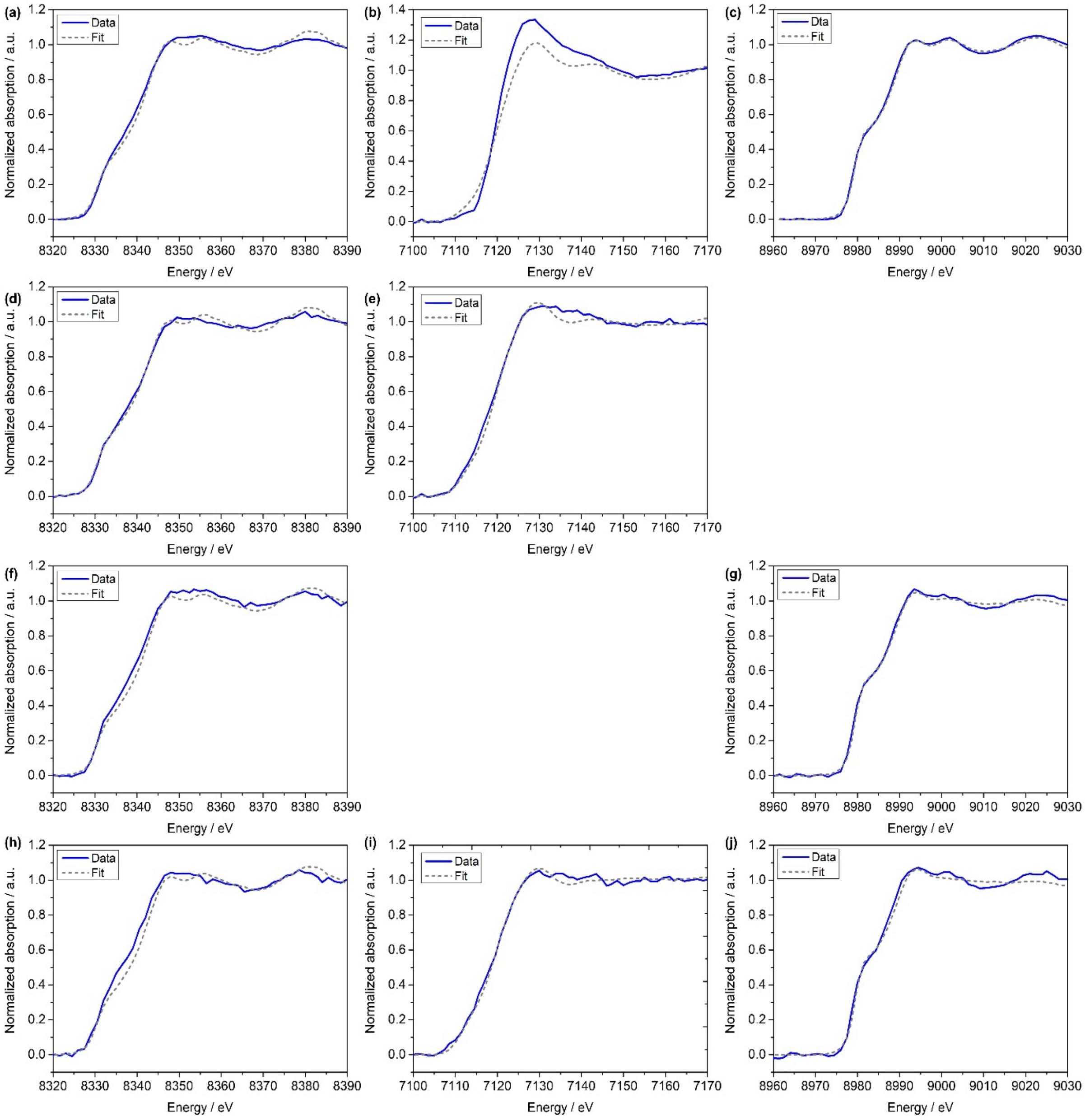

Figure S25. Linear combination fitting (LCF) of the X-ray absorption near-edge structure (XANES) at 400°C during CO_2 hydrogenation at the Ni (a, d, f, h), Fe (b, e, i), and Cu (c, g, j) K edge edge for the mono- (a-c), bi- (d-g), and trimetallic (h-j) catalyst materials.

Concerning the small nanoparticles (< 2 nm) supported on SiO_2 for all catalyst materials, it is expect that LCF yields slight deviations between the experimental XANES and the fit when considering bulk metal(oxide) reference compounds. However, this is a general issue for small nanoparticles which holds not only for laboratory- but likewise for synchrotron-based studies. The comparison of the LCF at 400°C during CO_2 hydrogenation with the corresponding metal foils (Figure S26) clarifies the differences between the experimental XANES and the metal foils. Especially for the XANES at the Fe and Cu K edge, it is evident that the XANES of the catalysts possess a higher white line intensity and different spectral features behind the absorption edges, indicating the contribution of oxidic species. Accordingly, the resulted contribution of metal oxidic species at 400°C during CO_2 hydrogenation is based on this deviation from metallic species rather than an artificial compensation of the broadened metal spectra due to the nanoparticle size of the catalyst materials during LCF. Moreover, to exemplarily illustrate the contribution of iron oxidic species for the monometallic Fe/SiO_2 catalyst at 400°C during CO_2 hydrogenation, the XANES of the catalyst is compared to those of the considered reference compounds from LCF (Figure S27). It is evident that the XANES of the monometallic Fe/SiO_2 catalyst shows characteristic features of the oxidic reference compounds, the white line intensity is significantly higher compared to the metal foil, and the absorption edge is shifted towards higher energies compared to the metal foil. During LCF, a combinatorial fit procedure is allowed, where all possible reference

compounds are entered but only the relevant ones were considered. The resulted combinatorial fit results for all possible combinations for the monometallic Fe/SiO_2 catalyst are additionally summarized in Table S4 and showing that the combination of FeO and Fe_3O_4 yields the best fit results. The presence of metal oxidic species during CO_2 hydrogenation is in accordance with the recently reported results for Co-based catalysts, where CoO was identified as catalytically active phase[6] and the results from XAS and XRD for Ni-Fe bimetallic systems [1, 7-9] showing the presence of Fe oxidic species both after reduction and under CO_2 hydrogenation conditions.

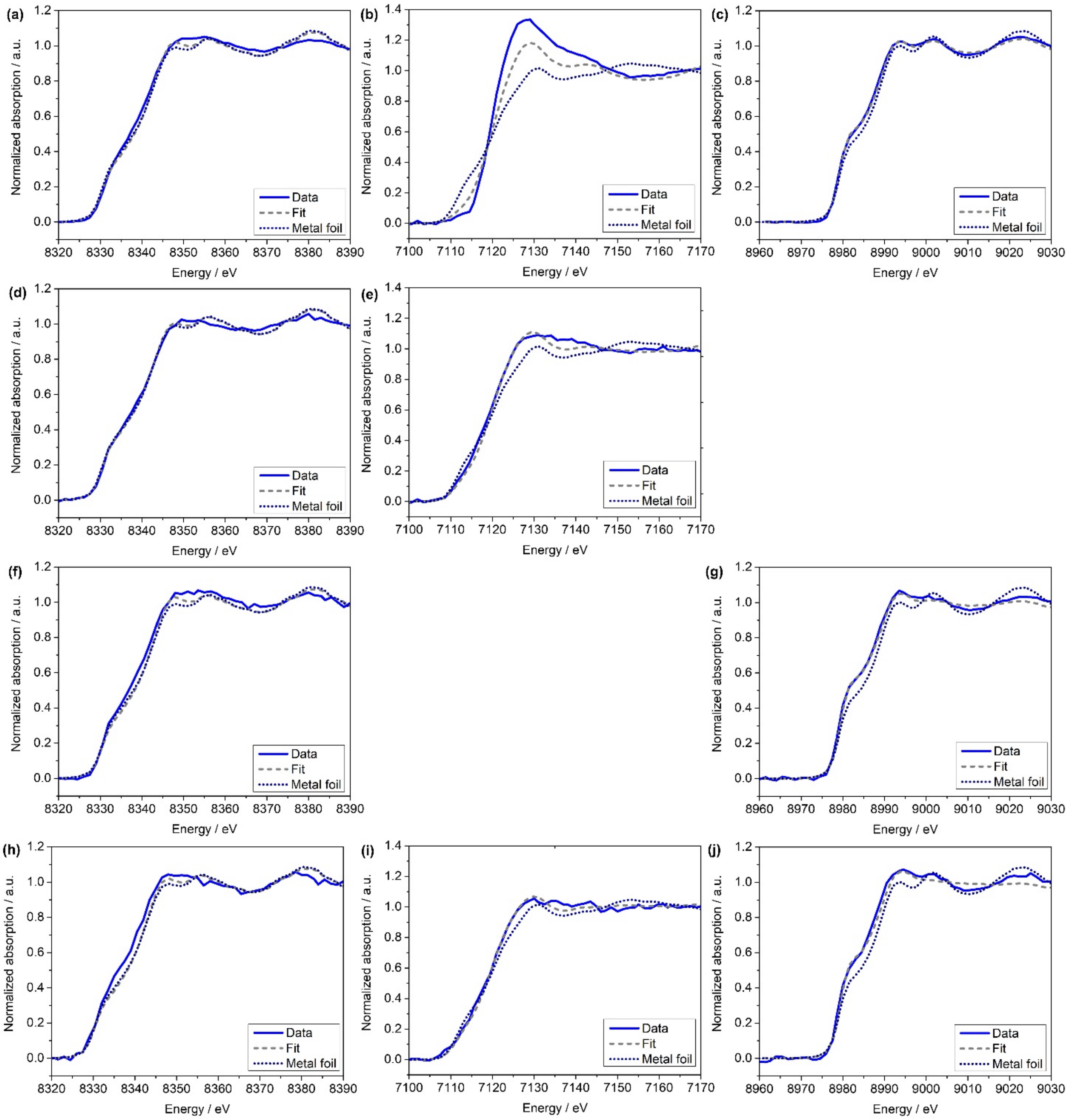

Figure S26. Linear combination fitting (LCF) of the X-ray absorption near-edge structure (XANES) at 400°C during CO_2 hydrogenation at the Ni (a, d, f, h), Fe (b, e, i), and Cu (c, g, j) K edge for the mono- (a-c), bi- (d-g), and trimetallic (h-j) catalyst materials together with the corresponding metal foils.

Table S4. Results from linear combination fitting (LCF) of the X-ray absorption near-edge structure (XANES) data obtained at the Fe K edge at 400°C during catalytic CO_2 hydrogenation for the monometallic Fe/SiO_2 catalyst using the combinatorial fit approach.

Combinations	R-factor	Weight A (Fe_2O_3)	Weight B (Fe_3O_4)	Weight D (FeO)	Weight C (Fe^0)
B, D	0.0629	-	0.252±0.092	0.748±0.092	-
A, B, D	0.0630	9.93E-09±0.289	0.233±0.321	0.767±0.441	-
B, C, D	0.0634	-	0.301±0.098	0.699±0.202	1.36E-08±0.132
A, D	0.0729	0.106±0.089	-	0.894±0.132	-
C, D	0.0757	-	-	1.000±0.132	1.01E-08±0.155
A, C, D	0.0780	0.250±0.110	-	0.750±0.237	3.26E-09±0.188
A, B, C, D	0.0910	5.39E-08±0.785	0.437±1.019	0.412±0.392	0.152±0.408
A, B, C	0.1174	-0.567±0.466	1	-	0.567±0
B, C	0.1550	-	0.690±0.667	-	0.310±0.142
A, B	0.1751	5.63E-08±0.466	1.000±0.802	-	-
A, C	0.2175	0.456±0.181	-	-	0.544±0.403

Additionally, to illustrate the contribution of iron oxidic species in the monometallic Fe/SiO_2 catalyst at 400°C during CO_2 hydrogenation, the XANES of the catalyst is compared to those of the used reference compounds from LCF. It is evident that the XANES of the monometallic Fe/SiO_2 catalyst shows characteristic features of the oxidic reference compounds, the white line intensity is significantly higher compared to the metal foil, and the absorption edge is shifted towards higher energies compared to the metal foil (Figure S27).

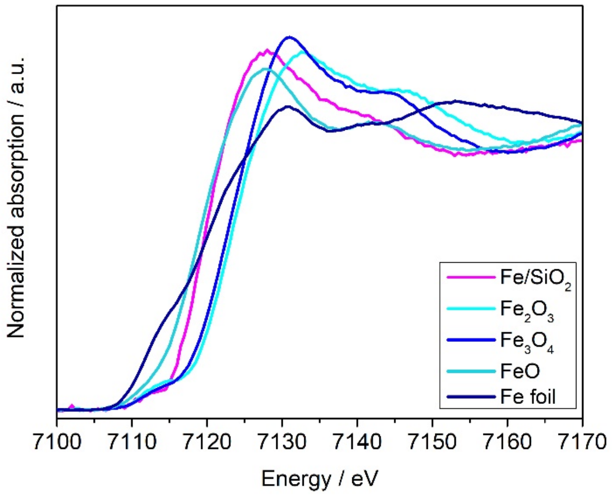

Figure S27. Comparison of the X-ray absorption near-edge structure (XANES) at 400°C during CO_2 hydrogenation of the monometallic Fe/SiO_2 catalyst with the used reference compounds from linear combination fitting (LCF).

Comparison between Reduction and CO_2 Hydrogenation

Based on the comparison of the XANES of the final reduced state at 520°C during reduction and those during catalytic CO_2 hydrogenation at 400°C for the multi-metal catalyst materials and corresponding monometallic counterparts, it is evident that there is no significant spectral change, i.e., all metal species show the same state as in the final reduced state (Figure S28).

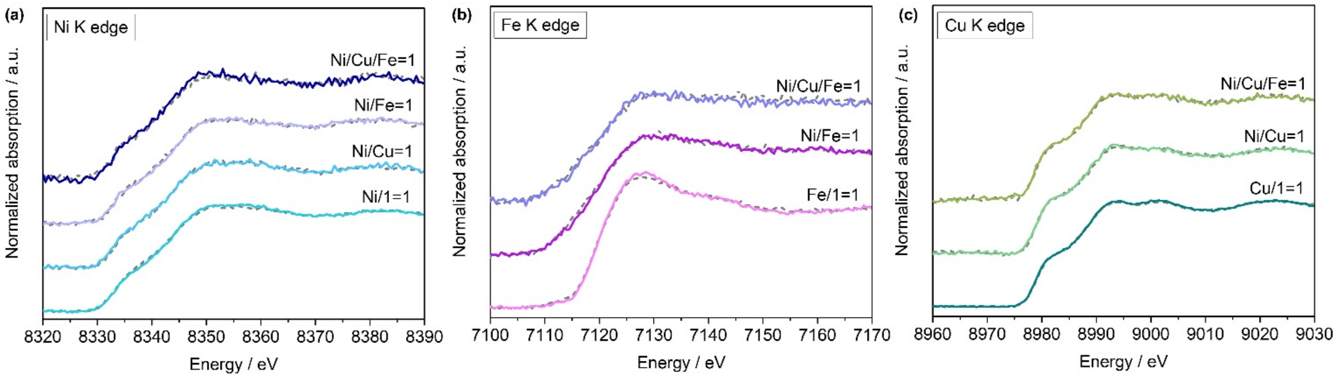

Figure S28. Comparison of the X-ray absorption near-edge structure (XANES) at the Ni (a), Fe (b), and Cu (c) K edge for the mono-, bi-, and trimetallic catalyst materials of the reduced state at 520°C (grey dotted line, H_2:He = 1:1, 1 bar) and those during catalytic CO_2 hydrogenation at 400°C (H_2:CO_2:He = 4:1:5, 1 bar) as indication for the degree of re-oxidation.

Additional Catalytic Performance Data

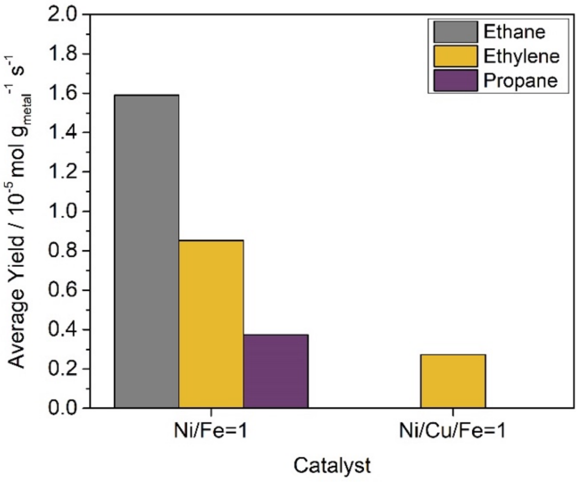

Figure S29. Average yield of C_{2+} products during catalytic CO_2 hydrogenation (H_2:CO_2:He = 4:1:5, 1 bar, GHSV = 4.1 10^4 h^{-1}) at 400°C for the bimetallic Ni-Fe/SiO_2 (Ni/Fe= 1) and the trimetallic Ni-Cu-Fe/SiO_2 (Ni/Cu/Fe=1) catalyst materials.

References

[1] L. R. Winter, E. Gomez, B. Yan, S. Yao, J. G. Chen, *Appl. Catal. B: Environ.* **2018**, *224*, 442–450.
[2] F. Krumeich, E. Müller, R.A. Wepf, R. Nesper, *J. Phys. Chem. C.* **2011**, *115*, 1080-1083.
[3] B. Ravel, M. Newville, *J. Synchrotron Radiat.* **2005**, *12*, 537-541.
[4] Institute for Catalysis, Hokkaido University “XAFS database”, can be found under https://www.cat.hokudai.ac.jp/catdb/index.php?action=xafs_dbinpbrowse&selection=2&r=13, **2022**.
[5] International X-ray Absorption Society, “IXAFS XAFS database”, can be found under http://ixs.iit.edu/database/, **2022**.
[6] I. C. ten Have, J. J. G. Kromwijk, M., Monai, D. Ferri, E. B. Sterck, F. Meirer, B. M. Weckhuysen, *Nat Commun.* **2022**, *13*, 324.
[7] K. Ray, G. Deo, *Appl. Catal. B Environ.* **2017**, *218*, 525–537.
[8] B. Jan, B. Zhao, S. Kattel, Q. Wu, S. Yao, D. Su, J. G. Chen, *J. Catal.* **2019**, *374*, 60–71.
[9] M. A. Serrer, A. Gaur, J. Jelic, S. Weber, C. Fritsch, A. H. Clark, E. Saraçi, F. Studt, J. D. Grunwaldt, *Catal. Sci. Technol.* **2020**, *10*, 7542–7554.

Author Contributions

N.S. Genz, A.-J. Kallio, and S. Huotari designed the operando XANES experiments, processed the raw data, and discussed the pre-processed data. N.S. Genz and A.-J. Kallio upgraded the XANES setup and performed the operando XANES experiments. R. Oord gave technical support during the operando XANES experiments. N.S. Genz and F. Meirer discussed the XANES data analysis and results and N.S. Genz analyzed the operando XANES data. F. Krumeich performed high-resolution (S)TEM at ETH Zurich and N.S. Genz performed the TEM analysis. U. Olsbye and Ø. Prytz enabled the contact to the TEM facility at University of Oslo and A. Pokle performed and analyzed the TEM measurements at the JEOL JEM-2100F microscope. N.S. Genz synthesized all catalyst materials and performed the XRD characterization. B.M. Weckhuysen directed the research. N.S. Genz wrote the manuscript under the supervision of B.M. Weckhuysen. All co-authors provided feedback to different rounds of revisions.
